# Recent Advances in the Application of Bionanosensors for the Analysis of Heavy Metals in Aquatic Environments

**DOI:** 10.3390/molecules29010034

**Published:** 2023-12-20

**Authors:** Bin Wu, Lu Ga, Yong Wang, Jun Ai

**Affiliations:** 1 College of Chemistry and Enviromental Science, Inner Mongolia Key Laboratory of Environmental Chemistry, Inner Mongolia Normal University, 81 zhaowudalu, Hohhot 010022, China; 15047091076@163.com; 2 College of Pharmacy, Inner Mongolia Medical University, Jinchuankaifaqu, Hohhot 010110, China; 13404832082@163.com; 3 College of Geographical Science, Inner Mongolia Normal University, 81 Zhaowudalu, Hohhot 010022, China

**Keywords:** bionanosensors, heavy metals, electrochemical bionanosensors, optical bionanosensors

## Abstract

Heavy-metal ions (HMIs) as a pollutant, if not properly processed, used, and disposed of, will not only have an influence on the ecological environment but also pose significant health hazards to humans, making them a primary factor that endangers human health and harms the environment. Heavy metals come from a variety of sources, the most common of which are agriculture, industry, and sewerage. As a result, there is an urgent demand for portable, low-cost, and effective analytical tools. Bionanosensors have been rapidly developed in recent years due to their advantages of speed, mobility, and high sensitivity. To accomplish effective HMI pollution control, it is important not only to precisely pinpoint the source and content of pollution but also to perform real-time and speedy in situ detection of its composition. This study summarizes heavy-metal-ion (HMI) sensing research advances over the last five years (2019–2023), describing and analyzing major examples of electrochemical and optical bionanosensors for Hg^2+^, Cu^2+^, Pb^2+^, Cd^2+^, Cr^6+^, and Zn^2+^.

## 1. Introduction

Heavy metals, along with other possible pollutants such as pesticides, home chemicals, and industrial compounds, are becoming increasingly important contributors to human health issues and environmental concerns. Heavy metals in the environment must be monitored for the following reasons: (1) Heavy metals are considered major environmental pollutants [1,2,3]. (2) Industrial production (e.g., mining and smelting), agricultural production (e.g., irrigation and fertilization), waste batteries, and electroplating all result in the gradual release of heavy-metal ions into the environment in the form of wastewater, waste gas, and waste residue [4]. (3) Heavy metals can accumulate through the food chain and other pathways due to their intrinsic incompatibility and difficulty in degradation, which has a substantial impact on the ecosystem and the human body. (4) There is a high risk of exposure to heavy metals in the human body, animals, plants, and elsewhere because of the way they change with the three-phase system of water, gas, and solid [5,6]. As a result, it is important not only to identify the sources and concentrations of heavy-metal ions (HMIs) but also to quickly and continuously monitor their levels in the environment.

Metals including sodium, potassium, magnesium, calcium, vanadium, chromium, manganese, iron, cobalt, nickel, cobalt, copper, zinc, and molybdenum are essential for human physiological functioning. Excess or deficiency of these critical elements in the human body, on the other hand, might have catastrophic consequences. Similarly, despite their extremely low concentrations, certain non-essential metals that enter the human body from various sources can have a major impact on human health status because they are not easily broken down and can easily accumulate in food [7]. Although some heavy metals, such as Fe, Se, Co, Cu, Mn, Mo, and Zn, are necessary for life, the majority of them are poisonous [8]. Mercury, for example, can cause considerable nervous system damage. Soluble mercury (Hg^2+^) is a highly toxic and bioconcentrated environmental pollutant. Mercury can be transformed into methylmercury, which builds up in the body and causes permanent injuries to the endocrine system, liver, kidneys, brain, and nervous system, among other disorders [9]. Lead (Pb) is found in automotive exhaust, old paint, mining waste incinerator ash, cigarettes, hair dyes, herbal treatments, and water in old lead pipes that need to be replaced, and lead exposure is detrimental to humans [10]. Lead ions can mimic essential ions (such as calcium, zinc, and iron) and hence affect the normal activity of crucial proteins. Affected proteins may be found in the cell membrane or cytoplasmic solutes of various tissues or systems [11]. In addition, lead both inside and outside of the cell disturbs the dynamic equilibrium of calcium, zinc, and magnesium plasma. The lungs, kidneys, bones, and immune system are all impacted by cadmium, a dangerous heavy metal that is used in mining, electroplating, and other sectors [1]. Lung cancer, prostate cancer, cardiovascular problems, and anemia can all result from this [12]. Trivalent chromium (Cr^3+^) is a trace element that is required by the human body [13]. In contrast, hexavalent chromium (Cr^6+^) is a very hazardous chemical that quickly enters the body, penetrates into cells, and kills them, causing harm to the organism [14]. Long-term and short-term hexavalent chromium exposure can result in ulcers, contact dermatitis, chronic bronchitis, gastrointestinal liver damage, emphysema, pneumonia, bleeding, liver and kidney damage, and tumors [15,16]. 

**Scheme 1:**
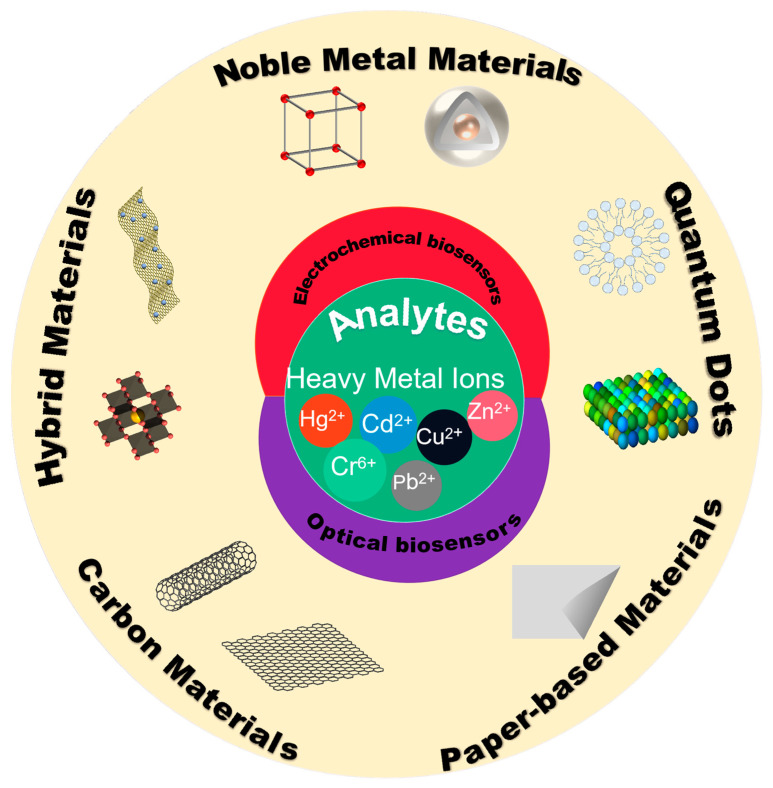
Progress in heavy metal detection methods.

As a result, determining necessary metal ions and non-essential metal ions in situ, in real time, and quickly is a pressing issue at the moment (Scheme 1). In this study, we will concentrate on biosensors for Hg^2+^, Cu^2+^, Pb^2+^, Cd^2+^, Cr^6+^, and Zn^2+^ ions.

## 2. Biosensors

According to the International Union of Pure and Applied Chemistry (IUPAC), ‘A biosensor is an integrated receptor sensor device capable of providing selective analytical information using biological recognition elements’ [17]. Notably, the suitable biocomponents and transduction module were chosen to make this biosensor sensitive and analyte selective, allowing for effective usage in toxicological research. The portable biosensor also allows for in situ analysis and real-time monitoring [18]. Bionanosensors are devices that can detect and measure small substances in the order of nanometers. Nanosensors take full advantage of their large specific surface area, high sensitivity, and specific chemical reaction properties to achieve fast and accurate detection of a wide range of contaminants.

The publication of the British journal Biosensing and the first conference on biosensing research in Singapore at the end of the twentieth century signaled the rise of biosensing research. This field’s development in China began more than 20 years later than it did elsewhere. The first national conference on industrial biochemical and enzyme engineering was conducted in China in 1986, initiating biosensing research [19]. Biosensors are made up of three components: a recognition element, a signal transduction element, and a signal amplification unit, with the recognition element consisting of antigens, antibodies, enzymes, aptamers, bacteria, cells, tissues, and so on [20]. Biosensors detect analytes with high sensitivity, quick detection speed, simple operation, low cost, and continuous dynamic monitoring by employing biologically active molecules as sensitive elements and chemical and physical conversion components. Biosensors have a high potential for development in medicine, biotechnology, the food business, and environmental pollution monitoring due to their unique benefits [21,22].

Atomic absorption spectrometry (AAS) [23], atomic emission spectrometry (AES) [24], inductively coupled plasma mass spectrometry (ICP-MS) [25], and cold vapor atomic fluorescence spectrometry (CVAFS) [26] are the most regularly utilized techniques for heavy metal identification. The methods described above have good sensitivity, selectivity, and accuracy. However, these systems have disadvantages, such as a difficult pre-treatment process, high operating costs, and the requirement for specialist people [3,27]. As a result, there is an urgent need for a measurement device that is simple to use, portable, and affordable. By contrast, biosensing technology offers numerous benefits and is steadily displacing traditional detection methods. In other words, the foundation of biosensors is simple and quick processing. The method is inexpensive, simple to use, practical, and only uses a small amount of chemicals. It can also be utilized as a portable field detection instrument because of its exceptional downsizing.

**Figure 1:**
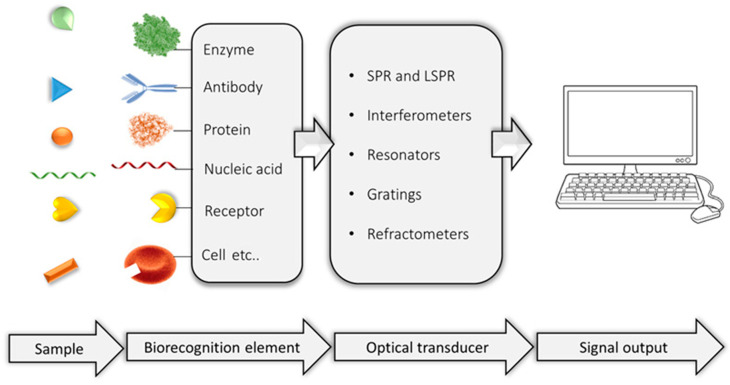
Optical biosensors [30]. Reprinted with permission [30]. Copyright 2016, Essays in biochemistry.

This paper summarizes the aspects of electrochemical biosensors and optical biosensors for the detection of heavy metals. Basically, an electrochemical sensor is a device capable of converting the reaction between a substance to be measured and an electrode into an electrical signal. An interaction takes place between the electrode and the electrolyte, which can be a direct reaction, such as the generation or use of ions or electrons, or an indirect reaction, such as changing the electrical properties of the electrolyte by changing its physical and chemical properties. This sensor detects the current and potential in the electrolyte and generates a corresponding electrochemical signal depending on the amount of substance measured. The signal is related to the amount of analyte of interest in the sample [28]. Optical biosensors are based on changes in optical properties, such as absorption, emission, transmission, and lifetime, caused by the interaction of the receptor with the substance to be measured [29]. Optical biosensors as shown in Figure 1 can use enzymes, antibodies, antigens, nucleic acids, whole cells, etc. as biorecording elements and detect the interaction between the biorecording element and the analyte using surface plasmon resonance (SPR), evanescent wave fluorescence, and optical waveguide interferometry [30].

## 3. Electrochemical Biosensor Detection of Heavy Metals

The advantages of the electrochemical approach include excellent sensitivity, selectivity, affordability, speed, portability, simultaneous detection of a number of target compounds, and environmental safety. It is a very promising detecting technique. Electrochemical sensors can be categorized into the following groups based on the biological recognition element they use: (a) DNA-based electrochemical sensors. These sensors use DNA as its biological recognition element. Electrostatic, groove-binding, or chimeric interactions between the drug to be tested and the DNA are possible. (b) Enzyme-based: in enzyme-based electrochemical biosensors, enzyme molecules serve as recognition components. By utilizing the great specificity of enzymes for the molecules to be detected, enzyme-based sensors can be created [31]. The electrochemical biosensor is a new detection method that combines the benefits of biosensing and electrochemical technology. It has a wide range of applications in food detection, environmental monitoring, medicine, clinical diagnosis, and therapy [32].

Heavy metals are typically present in their cationic form (Hg^2+^, Pb^2+^, Cd^2+^, et al.), allowing these heavy-metal ions to be electroreduced to their corresponding metals at the electrode. This process is analogous to a pre-enrichment process, in which even very small amounts of cations can deposit large amounts of metals at the electrode if the deposition (electroreduction) time is long enough. An anodic potential scan is then applied in a subsequent stage, leading the metal to be oxidized back to the matching cation [1].

Screen-printed carbon electrode (SPCE) electrochemical sensing technology, in particular, has the benefits of reusability, cheap cost, portability, customizability, and batch production, and has vast application prospects in the field of electrochemical sensing [33,34]. The SPCE electrode is a disposable electrode that consists of a working electrode, a reference electrode, and an auxiliary electrode [35]. This technology efficiently overcomes the issues associated with today’s expensive gold electrodes, the requirement for pre-treatment, the difficulty of regeneration after damage, and the interactions that occur while sharing one electrode for simultaneous assessment of numerous samples [36,37].

### 3.1. Mercury

**Figure 2:**
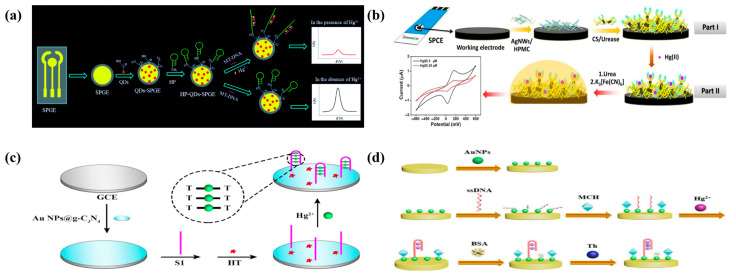
(**a**) Schematic illustration of the proposed HP-QDs-SPGE electrochemical biosensor [9]. Reprinted with permission [9]. Copyright 2022, RSC advances. (**b**) Schematic representation of the fabrication of Hg(II) biosensors based on the Ag-NWs/HPMC/CS/Urease-modified screen-printed carbon electrode [44]. Reprinted with permission [44]. Copyright 2021, Biosensors. (**c**) Schematic Representation of the PEC Biosensor for Hg^2+^ Detection [56]. Reprinted with permission [56]. Copyright 2022, ACS omega. (**d**) Preparation process of AuNPs/Au electrode, and schematic illustration of the fabrication procedure of the electrochemical aptasensor [58]. Reprinted with permission [58]. Copyright 2021, Anhui University.

Ono et al. [38] were the first to report that Hg^2+^ could bind preferentially to thymine (T) and form a more stable T-Hg-T aptamer. They then built the first DNA biosensor for detecting mercury ions by connecting a burst reagent on the end of a thymine fragment to a fluorophore. In the presence of Hg^2+^, the intensity of the redox reaction of the electrode to the signal indication decreases due to the conversion of flexible single-stranded DNA to rigid double-stranded DNA. The concentration of Hg(II) can be measured quantitatively and accurately using this method [39]. This principle still holds true today. Zhang et al. created a ratiometric hairpin-based DNA probe electrochemical biosensor for the detection of mercury ions by combining hairpin DNA probes with Zn-Ag-In-S quantum dots (QDs) and using screen-printed gold electrodes (SPGE) as a substrate (Figure 2a). When Hg^2+^ ions are present in large concentrations, the conformational hairpin DNA probe can be converted into a linear structure, resulting in a lengthy double strand at the QDs–SPGE interface and a weak electrochemical response. In the absence of Hg^2+^ ions, however, the hairpin DNA is unable to attach to the target molecule and so does not form a linear structure, resulting in a substantially greater electrochemical signal. The linear dynamic range of the technique is 10 pM–1 mM, and the detection limit is 0.11 pM. According to the application results, the developed electrochemical biosensor performs well in determining the mercury level of deionized water, tap water, groundwater, urine, and other materials [9].

Saenchoopa et al. created a disposable electrochemical biosensor for the detection of mercury ions in water by adapting a screen-printed carbon electrode (SPCE) as a template (Figure 2b). As shown in the figure, by surface-modifying the screen-printed carbon electrode, a composite layer of silver nanowires, hydroxymethyl propyl cellulose, chitosan, and urea (AgNWs/HPMC/CS/Urease) was created. Excellent electrical conductivity was achieved with the use of AgNWs, hydrophilic, degradable qualities with HPMC [40], and high loading properties with chitosan adhesive to improve the material’s sensitivity and stability [41]. Urease is immobilized on the sensing electrode, which causes urea to experience enzymatic digestion in the absence of mercury ions. By adsorbing ammonium ions and negatively charged Fe(CN)_6_^4−/3−^ at the same time, it is hypothesized that the AgNWS/HPMC/CS/modified electrode may acquire a positive charge, improving its ability to transport electrons [42,43]. According to earlier research, Hg(II) ions can generate inactive enzyme inhibitor complexes by forming covalent connections with the active centers of enzymes. As a result, there was a decrease in both the interaction of ferricyanide with the electrode and the generation of ammonia nitrogen (NH_4_^+^). Further investigation found that Hg(II) with pH variations causes the hydroxyl ion species (OH^−^) to resist the negatively charged Fe(CN)_6_^4−/3−^, limiting the capacity of the modified electrode for charge transport [42]. This biosensor had a sensitivity range of 5 to 25 M. The limit of quantification (LOQ) was set at 6.50 M, while the limit of detection (LOD) was 3.94 M. The accuracy of the biosensor was demonstrated by the agreement between the detection of mercury ions in water samples and the outcomes of inductively coupled plasma emission spectroscopy (ICP-OES) [44].

Based on Au-NPs@g-C_3_N_4_, which has excellent photoelectric capabilities, Li et al. developed a novel photoelectrochemical biosensor for the extremely sensitive and selective measurement of mercury ion (Hg^2+^) (Figure 2c). Graphite-like carbon nitride (g-C_3_N_4_) is favored due to its good chemical stability, easily tunable electronic structure, low cost, ease of availability, and lack of toxicity [45,46,47,48,49,50]. However, its photovoltaic properties are severely constrained by its rapid photogenerated electron-hole complexation and low specific surface. Due to the high specific surface area and superior optoelectronic characteristics of metal nanoparticles, which promote electron capture and transport, semiconductors treated with NPs can significantly enhance their optoelectronic capabilities. Furthermore, the presence of metal nanoparticles induces the surface plasmon resonance (SPR) phenomenon, which improves the capacity of semiconductors for charge transfer and light absorption [51,52,53,54,55]. As a result, the electrode surface of AuNPs@g-C_3_N_4_ was changed to provide it with good photoconversion capabilities, resulting in a greater initial photocurrent. Then, using Au-N bonding, thymidine-rich DNA was fixed on the surface of the modified electrode. To inhibit the non-specific binding sites, 1-hexanethiol (HT) was added to the resultant electrode. When the target Hg^2+^ is present, the selective capture of the target Hg by its base is used to generate a thymine–Hg^2+^–thymine (T-Hg^2+^-T) structure, which significantly reduces photoelectric conversion efficiency. The sensor has a large linear range from 1 pm to 1000 nm and a detection limit of 0.33 pm, allowing for highly sensitive mercury ion determination. Importantly, this research offers a novel approach for detecting Hg^2+^ and establishes a suitable platform for detecting other heavy-metal ions [56].

Hasanjani et al. used square-wave anodic solvation voltammetry (SWASV) to detect Hg using a pencil-type graphite electrode (PEG) modified with DNA/poly-L-methionine gold nanoparticles. The biosensing platform was exposed to the analyte solution and biased at 0 V for 250 s, while Hg^2+^ was tightly attached to thymine base (T) residues to form T-Hg^2+^-T complexes again. The current was then swept at 20 Hz from −0.50 to +0.60 V to promote Hg^2+^ reduction and reoxidation while recording SWASV to detect analytes in the 0.1 aM to 0.1 nM concentration range with a significant LOD of 0.004 aM [57].

Ma employed gold nanoparticles (AuNPs) as the working electrode to perform differential pulse voltammetry (DPV) analysis of mercury (Hg^2+^) utilizing electrochemical techniques (Figure 2d). The AuNPs were first generated by electrodeposition on the surface of the flat Au electrode, and then, the ssDNA was immobilized on the surface of the modified electrode by Au-S covalent bonding. T-base- and Hg^2+^-specific recognition occurred on ssDNA immobilized on the electrode surface, and parallel straight-stranded ssDNA self-folded and changed into a hairpin shape. An electrochemical indicator called thionine (Th) can then be specifically applied to the ssDNA bending structure, and Hg^2+^ can be detected based on the response of the Th differential pulse voltammetry signal [58].

Using the conducting polymer polyaniline-co-o-aminophenol (PANOA), Narouei et al. created a sensor with Au nanoparticles homogeneously scattered on its surface and strong recognition of Hg^2+^. The nitrogen functional groups of PANOA in combination with Au nanoparticles were found to have good adsorption on Hg(II) ions, which improved the detection efficiency of the sensor. The combination between PANOA fibers and Au nanoparticles resulted in highly sensitive detection of Hg. By SWASV analysis, the material yielded a lower detection limit of 0.23 nM for the detection of Hg^2+^ ions. The sensor was successfully employed to determine Hg(II) levels in water and fish samples [59].

### 3.2. Copper

In a multi-step process, Atapour et al. created a transparent conductive biosensor platform based on a sandwich structure of L-cysteine and gold nanoparticles to assess the concentration of Cu^2+^ ions in water. The limit of detection (LOD) and total linear range of the nanobiosensor were, respectively, 10–100,000 nM and less than 5 nM. A real sample of tap water was used to determine the trace Cu^2+^ levels using the developed nanobiosensor [60].

### 3.3. Lead

When interacting with Pb^2+^, the guanine (G)-rich oligonucleotide chain known as the Pb^2+^ aptamer can form a four-stranded structure [61,62,63].

**Figure 3:**
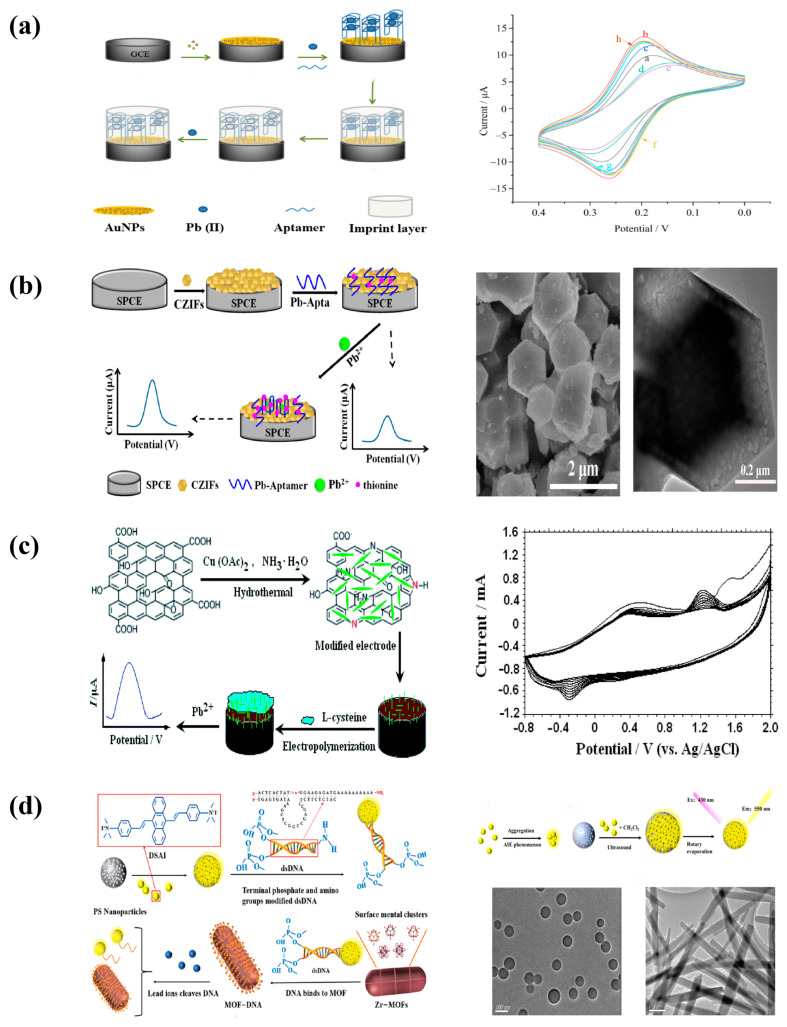
(**a**) Schematic diagram of aptamer-IIP-Pb(II) preparation for detection of Pb(II), typical CV curves of bare GCE, AuNPs/GCE, aptamer/AuNPs/GCE, aptamer/MCH/AuNPs/GCE and Pb(II)/Aptamer/MCH/AuNPs/GCE [64]. Reprinted with permission [64]. Copyright 2022, Molecules. (**b**) Presentation diagram of the preparation process for the aptasensor and Pb^2+^ detection, SEM image and TEM image of ZIF-8@ZIF-67 [65]. Reprinted with permission [65]. Copyright 2020, Biosensors. (**c**) The constructing process of the L-Cys/NN-CuO/N-rGO-modified electrode, electro-polymerized process of L-cysteine on the prepared NN-CuO/N-rGO/CPE by cyclic voltammetry for 10 cycles at the scan rate of 0.1 mV s^−1^ [66]. Reprinted with permission [66]. Copyright 2020, RSC advances. (**d**) Schematic diagram of AIEgen nanosphere-labeled aptamer combined with Zr-MOFs for detection of Pb^2+^, TEM image of AIEgen NPs and TEM image of MOF [67]. Reprinted with permission [67]. Copyright 20202, Biosensors.

Gold nanoparticles and aptamer were used by Zhu et al. to change the glassy carbon electrode, with the aptamer-imprinted polymer serving as the recognition component. Pb(II) caused the nucleic acid aptamer to adopt a G-quadruplex conformation in the presence of Pb^2+^, which would bind to particular spots on the aptamer-imprinted polymer (Figure 3a). The lead ions that are enriched on the working electrode decrease when the voltage is scanned from positive to negative, which causes a change in the current signal measured by the DPV method. The lead ion enrichment and current signal value increase with increasing lead ion concentration in the solution. Thus, by detecting the current signal value, the lead ion concentration can be determined [64].

In order to quickly determine the presence of Pb in water and contaminated soil, Ding et al. announced the development of carbon materials (CZIF) based on imidazolium core–shell molecular sieve as a framework (ZIF8@ZIF67) (Figure 3b). Strong aptamer binding ability is achieved by the high electrochemical activity, good stability, and high specific surface area of nanoporous carbon materials generated from a core–shell imidazolite framework. The creation of G-quadruplexes in nucleic acid aptamer molecules and the Coulomb interaction with thiocoronate caused by the addition of Pb^2+^ increase the electrical conductivity of these molecules. The current increases with Pb^2+^ concentration. The findings demonstrated a solid linear association between the Pb^2+^ concentration and the range of 0.1 to 10 ugL^−1^ with a detection limit of 0.096 ugL^−1^ [65].

Yang created a poly(L-cysteine)/CuO nanopins/N-doped reduced graphene oxide (L-Cys/NN-CuO/N-rGO) composite electrochemical sensor that is very sensitive and selective, and he used it to analyze trace lead ions (Figure 3c). L-cysteine was used in nature as a particular Pb^2+^ receptor, and NN-CuO/N-rGO nanocomposite was used as an enhanced sensing substance. Additional enrichment sites for Pb^2+^ are provided by the ability of cysteine to bind Pb^2+^. The electrochemical signal is enhanced and Pb^2+^ may be selectively recognized when L-Cys/NN-CuO/N-rGO interacts with Pb^2+^ analytes [66].

Using AIEgen nanoparticles as a fluorescent probe, Gao et al. created a nucleic acid aptamer sensor suited for determining lead ions in tea (Figure 3d). By altering the ends of the double-stranded DNA (-P) with phosphate groups (-P), the modified double-stranded DNA (dsDNA) was attached to the surface of Zr-MOFs. The other end of the Pb^2+^-specific cleavage site-containing DNA sequence was modified with an amino group (-NH_2_) to bind to carboxylated AIEgen NPS synthesized from polystyrene nanoparticles (PS NPS) and aggregation-induced luminescent material (DSAI) to form dsDNA with a fluorescent group (AIEgen-DNA).When dispersed in solution, AIEgen emits a mild luminescence, but when aggregated, it emits a bright fluorescence. The sensor was then treated with polyethylene glycol (PEG) to minimize interference from the substrate, increasing its sensitivity even further. Based on this, Zr-MOFs attached to dsDNA were used to quench the fluorescence of AIEgen nanoparticles (AIEgen NPS). The DNA sequence containing this cleavage site was cleaved and the conformation changed with the presence of Pb^2+^. To luminesce, AIEgen NPS and certain DNA sequences were extracted from the surface of Zr-MOFs. The concentration of Pb^2+^ was determined by comparing the fluorescence changes before and after the addition of Pb^2+^ [67].

### 3.4. Cadmium

**Figure 4:**
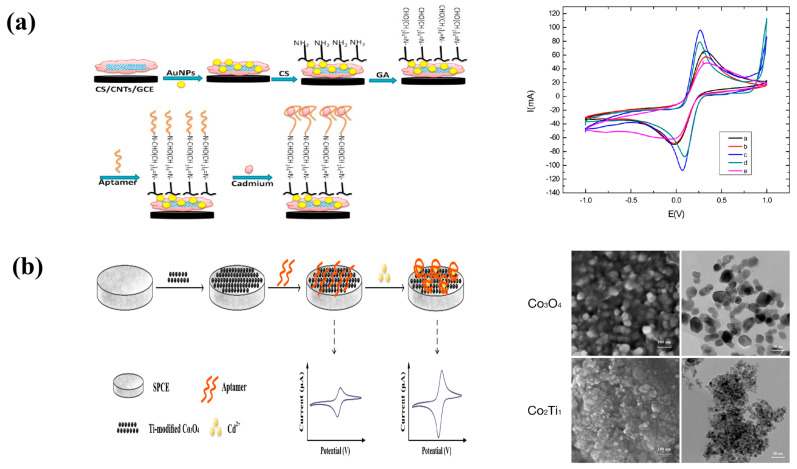
(**a**) Schematic illustration of GCE chemical surface modification with CNTs-AuNPs/CS nanocomposite for aptamer immobilization and application in cadmium detection, cyclic voltammograms in the presence of K_3_[Fe(CN)_6_]/K_4_[Fe(CN)_6_] for bare electrode (curve a), Cs-CNTs/GCE (curve b), AuNPs/Cs-CNTs/GCE (curve c), Apta/AuNPs/Cs-CNTS/GCE (curve d), Cd^2+^/Apta/AuNPs/Cs-CNTS/GCE (curve e) [72]. Reprinted with permission [72]. Copyright 2021, Sensors. (**b**) Schematic view of fabrication and principle of a label-free electrochemical aptasensor used for Cd^2+^ determination in the current study, SEM and TEM images of Co_3_O_4_ and Co_2_Ti_1_ [74]. Reprinted with permission [74]. Copyright 2020, Biosensors.

Rabai et al. created a label-free adaptive sensor to detect cadmium, a heavy metal, in water (Figure 4a). As can be seen in the figure, on a glassy carbon electrode, nanocomposites comprising carbon nanotubes, gold nanoparticles, and chitosan were cross-linked using an aptamer. Gold nanoparticles have a broad electrochemical potential range and improved electron transport, increasing the sensitivity of the biosensor. Chitosan increases their biocompatibility and solubility [68]. They boost biomolecule contact surface and grafting density, reducing diffusion issues. Furthermore, they preserve biomolecule stability and bioactivity [69,70]. The synergistic effect of carbon nanotubes and gold nanoparticles will allow for quicker electron transfer while maintaining strong electrical conductivity and electroactive surface area, resulting in a biocompatible milieu for the aptamer. The amino-modified aptamers and CS were then cross-linked with glutaraldehyde to the AuNP/CNT/CS-modified surface to immobilize the biometric elements [68,71]. The label-free adaption sensor detected cadmium with a detection limit of 0.02 pM [72].

Attaallah et al. investigated the first immobilized enzyme-based immunokinetic membrane to detect Cd^2+^ in drinking water as a colorimetric and electrochemical biosensor at the ppt/ppb level. Horseradish peroxidase (HRP) immobilization on the surface of immunokinetic membranes not only avoids the time-consuming immobilization method but also preserves the original enzyme structure for optimal function. The proposed enzyme membrane can precisely quantify Cd^2+^ in water samples without considerable interference from high Cu^2+^ concentrations due to dielectric exchange technology. When Cd^2+^ is present, the current increases dramatically in the HRP cyclic voltammetry, indicating that cadmium ions impede specific enzyme activities. Furthermore, the fast construction method, low price, controllability, easy control of enzyme loading, and long shelf life of the enzyme membrane have made it a popular application. Finally, this method has a lot of potential as a new in situ Cd^2+^ measuring tool, especially in resource-constrained scenarios [73].

Using Ti-modified Co_3_O_4_ nanoparticles, Liu et al. created a sensitive electrochemically aptamer-based Cd^2+^ detection sensor (Figure 4b). As a current signal amplifier, Ti-modified Co_3_O_4_ nanoparticles modified on the surface of a screen-printed carbon electrode (SPCE) are used, and as a probe, a nucleic acid aptamer for Cd^2+^ is used. Furthermore, sensing is based on an increase in the electrochemical probe thionine current signal as a result of particular detection of Cd^2+^ binding. Cd^2+^ can be determined in a linear range of 0.20 to 15 ng/mL under ideal conditions, with a detection limit of 0.49 ng/mL [74].

### 3.5. Chromium

Wu et al. created a photoelectrochemical (PEC) sensor based on bismuth vanadate (BiVO_4_) for very sensitive hexavalent chromium (VI) detection. BiVO_4_-7 has a high visible region energy band and a high specific surface with a wide band gap, high stability, and low toxicity [75], and is a new photoelectric detection material with great promise for applications [76], and is a potential nanomaterial for the photoelectric detection of heavy metals Cr(VI). Because of its low electron mobility, fast photocarrier complexation, and poor adsorption properties, bare BiVO_4_ has weak photocatalytic activity [77]. By manipulating the morphology, this study produced BiVO_4_ with carnation-like morphology and good optoelectronic characteristics. Because the conversion of Cr(VI) to Cr(III) speeds electron transmission, the photocurrent increases dramatically with increasing Cr concentration. It has been demonstrated to have a low detection limit (10 nM) and a broad detection range (2–210 M) [78].

Because of its capillary aspiration capabilities, paper is utilized as a substrate for immobilizing enzymes. It is lightweight, affordable, and can be easily patterned or sliced to meet the criteria of producing biosensors [79]. Dabhade et al. investigated the immobilization of glucose oxidase (GOx) on filter paper utilizing three polysaccharides as carriers, namely, chitosan, sodium alginate, and dextran, as well as screen-printed carbon electrodes for current measurement, and created paper-based electrochemical biosensor strips. By assessing the degree of enzyme inhibition, sensor activity, and lower limit, inhibition-based enzyme sensors identify the concentration of inhibitor in the sample under examination. Cr(VI) has the capacity to inhibit GOx Enyme [80]. Cr(VI) has a linear range of 0.05–1 ppm and a detection limit of 0.05 ppm [81].

### 3.6. Zinc

**Figure 5:**
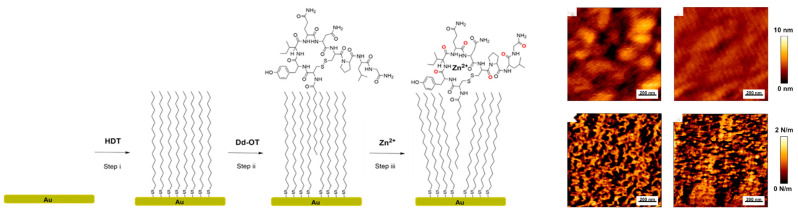
The fabrication steps of the sensing platform. Step i, self-assembly of HDT monolayer. Step ii, intercalation of Dd-OT molecule in the HDT SAM. Step iii, OT-Zn^2+^ complexation. Influence of zinc ion binding on the morphology and the stiffening of the layer: AFM topography analysis of HDT-Dd-OT (Ra = 1.05 ± 0.09 nm), HDT-Dd-OT-Zn^2+^ (Ra = 0.52 ± 0.05 nm), and slope of force–distance images of HDT-Dd-OT and HDT-Dd-OT-Zn^2+^ [84]. Reprinted with permission [84]. Copyright 2021, Scientific Reports.

Oxytocin (OT) is a neuropeptide with Zn^2+^ and Cu^2+^ ion affinity. Zn^2+^ chelation in OT happens via interactions with amide carbonyl groups [82,83]. Through contact, Attia et al. created a novel Zn^2+^ ion-selective receptor based on alkyl-amidated OT embedded in a subject alkanethiol monomolecular membrane (Figure 5). This insertion into the peptide monolayer causes an increase in the impedance response to ion binding. Its reaction to Zn^2+^ causes conformational changes, which result in morphological changes throughout the monomolecular layer. These changes were detected using impedance measurements, resulting in a selective Zn^2+^ ion sensor with a wavelength range of 0.1 nm to 10 nm. As a result, the monolayer serves as a host for the Dd-OT sensing peptide, enhancing RCT proliferation of biometric events. This method differs significantly from sensors made from directly covalently bonded OT monolayers. This non-covalent construction method can be expanded to various bioreceptors embedded in natural and artificial membranes [84].

## 4. Optical Biosensor Detection of Heavy Metals

Optical biosensing can be divided into two categories: markerless and marker-based. In short, the detection signal is created directly by the interaction of the substance being studied with the transducer in the label-free mode. Marker-based sensing, on the other hand, comprises the employment of a marker followed by an optical signal created by colorimetric, fluorescent, or luminescent approaches. Optical biosensing technology will be used more widely in areas such as healthcare, biomedicine, and biomedical sciences. This will not only provide a further reduction in detection means but also aid in the screening of huge samples under many indications at high throughput and sensitivity [30].

Optical biosensors are classified as follows: surface plasmon resonance biosensors (SPR): a biosensor that emits polarized light at a specific angle of view onto the surface of a metal (or other conductive substance), generating an SPR effect. The SPR phenomenon allows the refractive index of the sensor surface to change directly, label-free, and in real time, in proportion to the concentration of biomolecules [30]. Localized surface plasmon (LSP)-based biosensors: when the SP is contained in nanoparticles (NP) of size comparable to the incident wavelength and the free electrons of the particles experience collective oscillations, the resulting effect is known as LSP. The LSP is a nonpropagating SP that is concentrated in a small area close to the particle surface, and the size, shape, and composition of the particle can alter its resonance [85]. PC-based biosensors (PC-based biosensors): a PC is made up of two or more materials with different refractive indices that are organized in space on a regular basis and manipulate light throughout a wide range of optical wavelengths [86]. Photons, like electrons, can be thought of as having a band structure in PC structures. Optical-fiber-based biosensors: optical fibers are made up of a core and a cladding that has a refractive index slightly higher than the cladding to direct incident light. To protect the fiber, an extra layer is added as a secondary cladding [87]. Light propagates via an optical cable according to Snell’s law of reflection. As a result, optical fibers are frequently referred to as ‘optical waveguides’. Because of its resistance to external electromagnetic interference and capacity to live in severe settings and extreme temperatures, fiber-optic sensors have received a lot of interest in several analytical domains.

Fluorescence approaches for sensing have several advantages. First, molecular fluorescence is extremely sensitive; these measurements cause little or no harm to the host structure, allowing for entirely non-invasive sensing. Second, fluorescence techniques reveal information about the system design and environmental behavior of molecules, as well as how these molecules respond to changes in analytes in their surroundings. Some heavy-metal-sensitive probe-labeled proteins, for example, can be quenched or increased by adaption modifications indicated by dye heavy metals. Fluorescence resonance energy transfer (FRET) can be used to study the distribution of biomolecules in specified habitats and under such conditions [88,89].

### 4.1. Mercury

There are now two major categories of investigations focusing on fluorescence probes for mercury ions. One approach is to use Hg^2+^ complexation to modulate fluorescence intensity [90,91,92], but the fluorescent molecules produced by this approach are susceptible to interference from other metals (e.g., Cu^2+^, Co^2+^, Fe^2+^, Pb^2+^ [93,94,95,96]) or fluorescence bursts caused by the heavy atom effect induced by Hg^2+^ ions [97,98,99,100]. Another approach is to use mercury ions to specifically recognize fluorescent compounds [101,102,103,104,105]. When compared to chelation procedures, the unique spectrum changes in fluorescence or absorbance caused by mercury ions make this method more selective and sensitive. This method, however, may occasionally be hampered by aggregation-caused quenching (ACQ) effects generated by the aggregation of reaction products having hydrophobic characteristics [106,107]. With the advent of the aggregation-induced emission (AIE) phenomenon, there is now a chance for a breakthrough in this challenge [108]. Despite the fact that a number of mercury ion probes with AIE luminescence mechanisms have been reported, the development of efficient and sensitive AIE-emitting mercury ion probes remains a current research priority.

Tang et al. proposed the TPE-M fluorescent probe, produced from tetraphenylene, for the detection of Hg^2+^ in CH_3_OH/PBS (20 mM, pH 1.4–7.4) (3:7, *v*/*v*) solutions. When the non-emitting probe TPE-M solution is treated with Hg^2+^, the dithiocarbon hydrolyzes, releasing its precursor 4 (a possibly AIE-active molecule) and causing considerable fluorescence amplification, enabling for Hg^2+^ detection in aqueous conditions. This Hg^2+^ identification event features a quick response time, excellent selectivity and sensitivity, good anti-interference capability, and a low detection limit. TPE-M may also be used to detect Hg^2+^ in real food samples such as shrimp, crab, and tea, which increases its usability [109].

Fan et al. developed a mercury ion (Hg^2+^) proportional fluorescent probe based on nitrogen-doped blue carbon dots (NCDs) and red gold nanoclusters (AuNCs) for rapid visual and quantitative detection and applied it to a fluorescent test paper-based sensor. Hg^2+^ can, in particular, attach to Au^+^ on the surface of AuNCs, causing a red fluorescence burst, whereas nitrogen-doped AuNCs emit a blue fluorescence burst. As an internal standard signal, the fluorescence intensity of blue carbon dots remains constant. Thus, fluorescent colors ranging from red to pink, pink to purple, and purple to blue can be easily recognized visually and statistically studied under UV light using a smartphone. The use of paper-based sensors eliminates common analysis process limitations such as detection time and analysis expense. In a subsequent demonstration, Hg^2+^ was determined using a handmade smartphone detection gadget. After adding varying quantities of Hg^2+^, the detecting area of the fluorescent test paper turned red, purple, and blue. Color mapping in the Android app was used to determine the red, green, and blue (RGB) values of fluorescent colors. Hg^2+^ detection is quick and quantitative, with a fluorescence detection limit of 2.7 nM, a smartphone detection limit of 25 nM, and a paper strip detection limit of 32 nM. With satisfactory recovery, the devised multimode detection platform was successfully used in the detection of mercury ions in water samples. Individual on-site environmental monitoring of Hg^2+^ with the ‘naked eye’ and cellphones is made possible by the NCD and gold NCD probes [110].

Ali et al. developed a new chemical sensor for the detection of mercury (II) ions, the sensor film was based on 2-hydroxy-3((2S,2′R,3A′S,5R)-2-isopropyl-5,5′-dimethyl-4′-oxotetrahydro-2′H-spiro[cyclohexane-1,6′-IM-imidazo[1,5b]isoxazole]-2′-yl)methyl)-5-methyl based on methyl benzoate (IXZD). IXZD molecules are encapsulated in PVC films. The fluorescence peak at 399 nm rose dramatically during the fluorescence titration. The burst of the emission center peak at 481 nm demonstrated the chelating effect of the IXZD sensing probe on mercury ions. Furthermore, with the gradual increase in Hg(II), the emission peak at 475 nm was greatly strengthened. The detection limit was 0.025 mM, and no cation interfered [111].

Gupta et al. created whole-cell and cell-free biosensors for mercury detection. Biosensors were created using transgenic plasmids expressing merR genes. As reporter genes, the firefly luciferase (LucFF) and emerald green fluorescent protein (EmGFP) genes were used. EmGFP fluorescence was diminished in the cell-free system due to pH fluctuations and the bursting effect of mercury excess. EmGFP fluorescence was partially restored after the pH was lowered to 7 and chelators were applied. When utilizing this procedure, it was thought that the contamination itself could be detrimental to the biological system, causing mistakes. To circumvent this, a cell-free approach was devised, which was thought to be a better alternative for assessing pollutants because it was not affected by the cellular reaction to the contaminant. Furthermore, by removing cell culture, the whole experiment time can be significantly decreased. Finally, it is possible to design merR gene-based biosensors for the detection of a wide spectrum of mercury pollutants in water using these creations and cell-free systems [112].

Kim et al. created whole-cell biosensors in E. coli using copA, zntA, and mer promoter plasmids for copper, cadmium, and mercury detection. The luciferase (lux) gene was added to the plasmid as a reporter gene, and the plasmid was then replaced with an optical detection fusion protein sequence incorporating OmpA (1–159) and mCherry. For Hg, Cd, and Cu, the linear ranges of the produced strains were 0.1 ppm, 0.2 ppm, and 27.5 ppm, with linear ranges of 0.99030, 0.99676, and 0.95933, respectively. Visual inspection of the strains revealed the presence of these three heavy metals [113].

Acha et al. demonstrated a fluorescent fiber-optic sensor for detecting mercury ions in aqueous solutions. A fluorophore-labeled thymine (T)-rich oligodeoxynucleotide (ON) sequence was mounted directly on the tip of a tapered optical fiber to form the sensor. The T-Hg^2+^-T mismatch generated in the presence of mercury ions attenuated the emitted luminescence, allowing Hg^2+^ ions to be determined in aqueous solutions [114].

**Figure 6:**
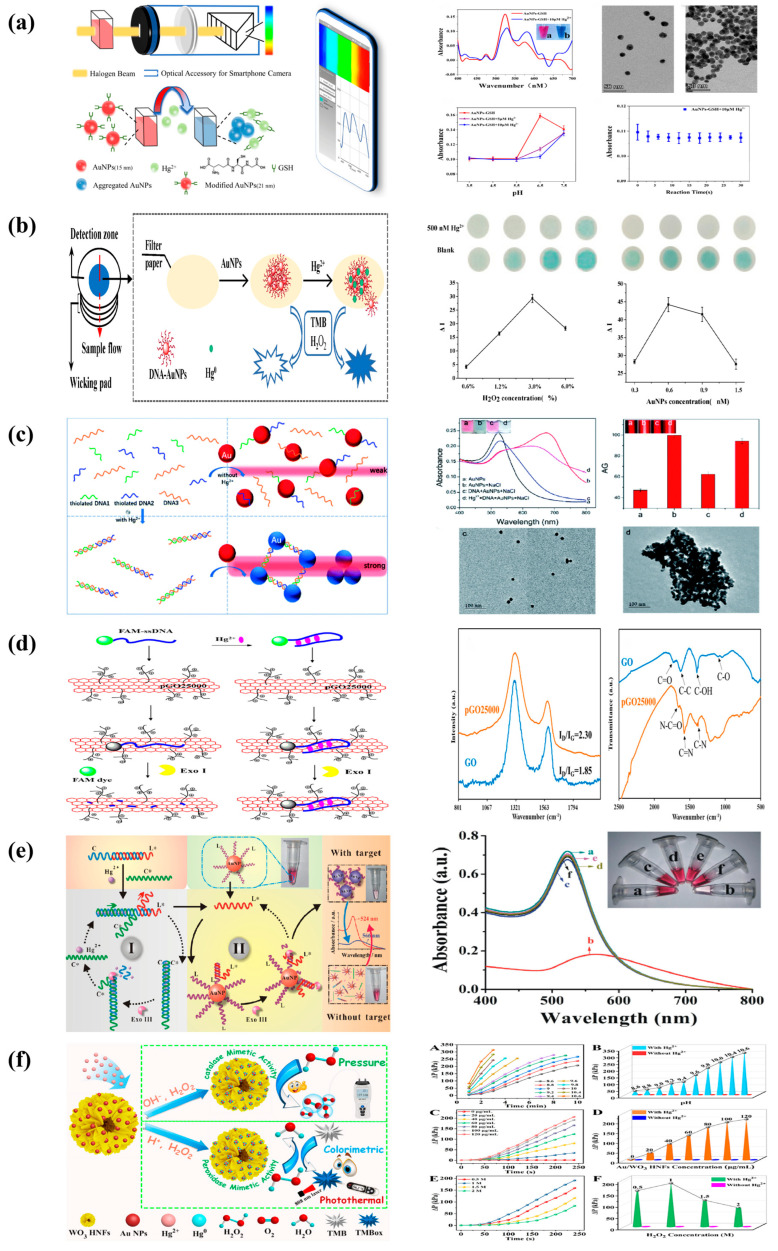
(**a**) Colorimetric biosensor system based on a smartphone and schematic illustration of Hg^2+^ detection based on AuNPs-GSH. Characterization of the absorption spectra of the AuNPs−GSH solution using the proposed sensor and TEM images of the AuNPs−GSH solution before and after addition of Hg^2+^ [120]. Reprinted with permission [120]. Copyright 2022, Biosensors. (**b**) Schematic diagram of Hg^2+^ detection. Optimization of H_2_O_2_ and DNA–AuNP concentration [121]. Reprinted with permission [121]. Copyright 2020, Biosensors. (**c**) Schematic representation of the novel TEA method with the bare AuNPs for the colorimetric detection of Hg^2+^ ions based on the specific T-Hg^2+^-T coordination chemistry. Transmission electron microscope (TEM) images and their average gray (AG) values measured [125]. Reprinted with permission [125]. Copyright 2021, RSC advances. (**d**) Schematic diagram of the designed fluorescence ‘turn-off’ strategy for Hg^2+^ detection with the assistance of Exo I nuclease using a FAM–ssDNA probe. Characterization of GO and pGO25000 by Raman spectroscopy and FT-IR [127]. Reprinted with permission [127]. Copyright 2022, International Journal of Molecular Sciences. (**e**) Working principle diagram of mercury ion colorimetric biosensor, UV absorption spectrum of mercury ion colorimetric biosensing strategy. The inset shows the corresponding color change of the solution [128]. Reprinted with permission [128]. Copyright 2019, Jinan University. (**f**) Schematic diagram of Hg^2+^ detection based on the pressure-sensing platform and colorimetric and photothermal sensing platform, pressure signal change profiles of H_2_O_2_ decomposition with reaction time under different pH conditions (A), different Au/WO_3_ HNFs concentrations (C), and different H_2_O_2_ concentrations (E). The pressure signal change values (ΔP) of H_2_O_2_ decomposition to produce O_2_ are dependent on the pH values (B), the concentration of Au/WO_3_ HNFs (D), and the concentration of H_2_O_2_ (F) in the absence and presence of Hg^2+^ [129]. Reprinted with permission [129]. Copyright 2022, ACS Applied Materials & Interfaces.

Gu et al. used nanogold–glutathione (AuNPs-GSH) to detect Hg^2+^ in water (Figure 6a), where the mobile sensor terminal combined with a smartphone camera and an optical setup to process the incoming images finally achieved the expected spectral detection with a detection limit of 1.2 nM. AuNPs have a high affinity for biothiols, and when molecules containing thiol groups (for example, mercury, Hg^2+^ has a higher sulfurophilicity [115]) are added to the AuNP solution, they quickly bind to the surface of the AuNPs [116], causing AuNP aggregation and a color change from red to blue [117,118,119]. This approach was also used to successfully recover natural mineral water, pure water, tap water, and river water, with recoveries ranging from 97.4 to 115.9% [120].

Mao et al. created a paper gadget from a patterned paper sheet, an absorbent pad, and a foundation (Figure 6b). DNA–gold nanoparticles (DNA-AuNPs) were deposited on the paper sheet, and Hg^2+^ ions could be absorbed by DNA-AuNPs. The generated DNA-AuNP/Hg^2+^ nanoparticles can catalyze the chromogenic reaction tetramethylbenzidine (TMB)-H_2_O_2_. Because of the effect of the absorbent pad, larger volumes of Hg^2+^ samples can be applied to paper-based devices for Hg^2+^ detection, enhancing the color reaction. This paper-based system obtains a cut-off value of 50 nM for Hg^2+^ detection by the naked eye under optimal conditions. Furthermore, quantitative measures can be obtained by scanning images using a desktop scanner and extracting grayscale data. With a detection limit of 10 nM, a linear range of 50–2000 nM Hg^2+^ was found. Furthermore, the paper-based device can be used on environmental water samples with recoveries ranging from 85.7% to 105.6%. The colorimetric assay based on the paper device has a significant potential for practical sample applications due to its low cost and simplicity [121].

Many nanomaterials, including gold nanoparticles (AuNPs), gold nanorods, and silver nanoparticles, can interact significantly with Hg^2+^. When these nanoparticles absorb Hg^2+^, they can create Au-Hg nanoalloys or Ag-Hg nanoalloys [122]. Several studies have shown that Au-Hg nanoalloys have peroxidase-like characteristics and can catalyze the H_2_O_2_-mediated oxidation of tetramethylbenzidine (TMB) [123], but the catalytic activity is low. Hg^2+^ can be trapped by DNA-AuNP complexes, resulting in DNA-Au-Hg nanoalloys [124]. The peroxidase activity of DNA-AuNPs is greatly increased upon Hg^2+^ adsorption, resulting in a very high TMB-H_2_O_2_ peak at 650 nm. It was possible to produce highly sensitive colorimetric detection of Hg^2+^.

Chen et al. created a novel nanosensor for the easy, rapid, and portable colorimetric detection of mercury (Hg^2+^) ions by combining the sensitive Tyndall effect (TE) of gold nanoparticles (AuNPs) with thymine–Hg^2+^–thymine (T-Hg^2+^-T) coordination chemistry (Figure 6c). This is the first report on the development of a TE-based biosensor for the measurement of highly hazardous heavy-metal ions employing nanoparticles and functionalized nucleic acids. Two short thiol ssDNAs (DNA1 and DNA2) were employed, as well as their long complementary ssDNA (DNA3). In the absence of Hg^2+^, these sufficiently soluble ssDNAs with partially uncoiled bases allow stable dispersion of unmodified AuNPs in high-ionic-strength solutions and self-assembly on the nanoparticle surface via gold thiol (Au-S) interactions and van der Waals forces, resulting in relatively weak TE. Short ssDNAs (i.e., 12-base DNA1 or DNA2) are especially well suited for adsorption on such bare nanoparticles. The analyte ions can induce the hybridization of the three ssDNAs via the T-Hg^2+^-T-specific ligand process to produce stable rigid double-stranded DNA (dsDNA) structures with the addition of Hg^2+^. Aggregation of AuNPs would result in a much amplified TE signal because they are separated not only by the terminal thiol groups at the ends of each dsDNA but also by a lack of sufficient labile ssDNAs to act as stabilizing molecules. After the addition of Hg, analyte ions can facilitate the hybridization of the three forms of ssDNA to create stable rigid double-stranded DNA (dsDNA) structures via the T-Hg^2+^-T-specific ligand interaction. As a result, AuNP aggregation occurs, resulting in a greatly amplified TE signal, because they are not only attracted closer to each other by the terminal thiol groups at the ends of each dsDNA but also lack sufficient flexible ssDNAs to act as stabilizing molecules. Because of their thiophilic character, residual Hg^2+^ ions are more likely to be retained by excess nonfunctionalized DNA3 and prevent the removal of thiolated oligonucleotides from the particle surface. A red laser pointer (635 nm) is used to induce the TE reaction, visible TE changes alone can be used to perform a qualitative or semi-quantitative examination of Hg^2+^ levels in the sample. The TEA approach requires only a low-cost laser pointer for the operator to achieve qualitative visual examination of 625 nm Hg^2+^ in less than 10 min [125].

Gao et al. showed a set of simple, low-cost, and highly sensitive graphene oxide GO/DNA hybridized biochips. The biochips were prepared from graphene oxide (GO) activated by NHS (n -hydroxysuccinimide)/EDC(1-(3-dimethylaminopropyl)-3-ethylcar -bodiimide salt) and DNA modified by phosphorothioate (PS) cleavage sites and Cy5 fluorophore, and the graphene oxide/DNA hybrids were further immobilized on the carriers to achieve high sensitivity for Hg^2+^ and high-throughput detection. The biochip’s optimal limit of detection (LOD) is 0.38 nM with a selectivity larger than 10:1. Physical adsorption and chemical bonding are the most common mechanisms by which GO interacts with DNA. Non-physically adsorbed DNA might generate background interference in the sensing device, resulting in an excessive number of false positive signals. Chemical ligation, on the other hand, is a time-consuming and expensive operation. While chemical bonding has an acceptable fixing rate, unlinked DNA can be removed using other means, such as centrifugation, with great interference resistance. The Cy5 fluorescent group on DNA is released into the reaction fluid after PS cleavage with Hg^2+^. The fluorescence intensity of the slide drops after the reaction solution is sucked away, according to the principle of fluorescence resonance energy transfer (FRET). The cutting rate increases as the Hg^2+^ concentration increases, whereas the fluorescence intensity of the corresponding slide decreases. As a result, the analytes can be measured by comparing the fluorescence intensity before and after the addition of Hg^2+^ [126].

Sun et al. created a water-soluble positively charged graphene oxide (pGO25000) fluorescence burst by carbodiimide grafting polyetherimide onto graphene oxide nanosheets (Figure 6d). The stronger electrostatic interactions created by pGO25000’s increased affinity for DNA strands were attributed to the much higher fluorescence bursting ability of pGO25000 compared to graphene oxide. A FAM-ssDNA probe was created employing pGO25000 as a fluorescent bursting agent for the extremely selective and ultra-sensitive detection of mercury ions under mild circumstances using the EXO I enzyme. When Hg^2+^ is present, the hairpin structure of the FAM-labeled single-stranded DNA probe becomes T-Hg^2+^-T, with no fluorescence. Exonuclease I digests the lack of Hg^2+^, restoring fluorescence. This sensor’s fluorescence intensity was favorably linked with Hg^2+^ concentration in the 0–250 nm range (R^2^ = 0.9955), with a detection limit of 3.93 nm [127].

Song created a highly sensitive and efficient recognition biosensor using the specific T-Hg^2+^-T structure of mercury ion (Hg^2+^) and DNA isothermal signal amplification technology (Figure 6e), which was successfully applied to the detection of trace heavy-metal ions in natural water bodies. The mercury ion colorimetric biosensor was created by promoting the aggregation and sinking of nanogold by activating the hydrolysis of nucleic acid exonuclease III via the chain substitution reaction produced by mercury ions. T-Hg^2+^-T operates as a recognition element, and the strand replacement reaction product caused by mercury ion specific binding generates a stable double strand with 3’ flat ends. The nucleic acid exonuclease III enzyme was activated, and the double-loop process formed by its hydrolysis reaction resulted in a significant reduction in small single strands of DNA modified on the surface of the nanoparticles, as well as a decrease in interparticle distance. This causes the reaction sample’s color to shift from red to blue, a red shift in the wavelength of the absorbed light, and a commensurate decrease in absorbance [128].

Zhi et al. constructed a novel, simple, label-free, and portable detection platform using hollow-WO_3_–hollow-nanoflower-modified with gold nanoparticles to achieve highly sensitive and selective Hg(II) analysis using three detection modes: pressure, temperature, and colorimetric. In this project, it is proposed to prepare hollow Au/WO_3_ nanoflakes (Au/WO_3_ HNFs) that can effectively prevent the agglomeration of Au nanoflakes and significantly improve their catalytic performance and stability. 1. In the Hg(II) mode, when analytes are present, the H_2_O_2_ in this detection system splits rapidly into O_2_, which generates detectable pressure signals. 2. Using Au/WO_3_ HNFs as a model for mercury to study their enzymatic activities under the excitation of mercury ions and test their colorimetric, photothermal, and other properties. In addition, the multi-mode detection system proposed in this project is effective in detecting Hg(II) in water bodies [129].

### 4.2. Copper

**Figure 7:**
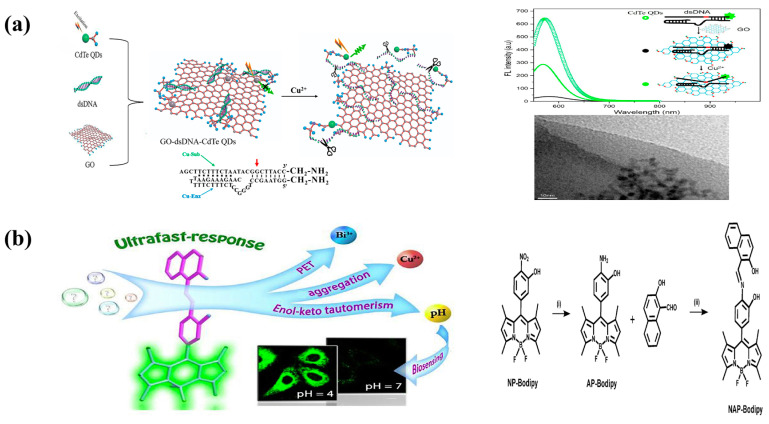
(**a**) Illustration of the operating principle of the ‘turn-on’ fluorescent sensor for Cu^2+^ detection. The FL spectra of GO-dsDNA-CdTe QD complex in the absence or presence of Cu^2+^ and TEM images of GO-CdTe nanocomposite [130]. Reprinted with permission [130]. Copyright 2018, Sensors. (**b**) The BODIPY molecular sensor exhibits ultra-fast detection of a wide range of analytes and serves as a good detector of lysosomes during pH changes in live HeLa synthesis of NAP-BODIPY [131]. Reprinted with permission [131]. Copyright 2019, Dyes and Pigments.

Ding et al. effectively produced a novel ‘turn-on’ fluorescent copper biosensor by chemical cross-linking graphene oxide (GO)-dsDNA-CdTe quantum dot (QD) complexes (Figure 7). Because of the surface and burst character of two-dimensional GO, the fluorescence burst of CdTe quantum dots is a FRET mechanism. The copper ion causes a catalytic reaction in the DNA strand, causing irreversible breakage at the cleavage site, resulting in the production of G-quadruplexes and the separation of CdTe QDs from GO and the restoration of its fluorescence. Thus, in the presence of copper ions, the fluorescence of GO-dsDNA-CdTe quantum dot complexes recovers significantly [130].

Chen et al. developed a class of sensors, NAP-BODIPY, consisting of a receptor (naphthol-azomethine-phenol) and a single fluorophore (BODIPY). The addition of copper ions to the NAP-BODIPY system resulted in a significant red shift of the maximum absorption peak from orange-yellow to violet-red due to metal-induced agglomeration. However, the presence of Bi^3+^ ions in the NAP-BODIPY system inhibited the photo-induced electron transfer process, resulting in a strong enhancement of the weak fluorescence emission. The addition of Cu^2+^ to the NAP-BODIPY-Bi^3+^ (1:10) system then led to a change in the triple signal [131].

### 4.3. Lead

**Figure 8:**
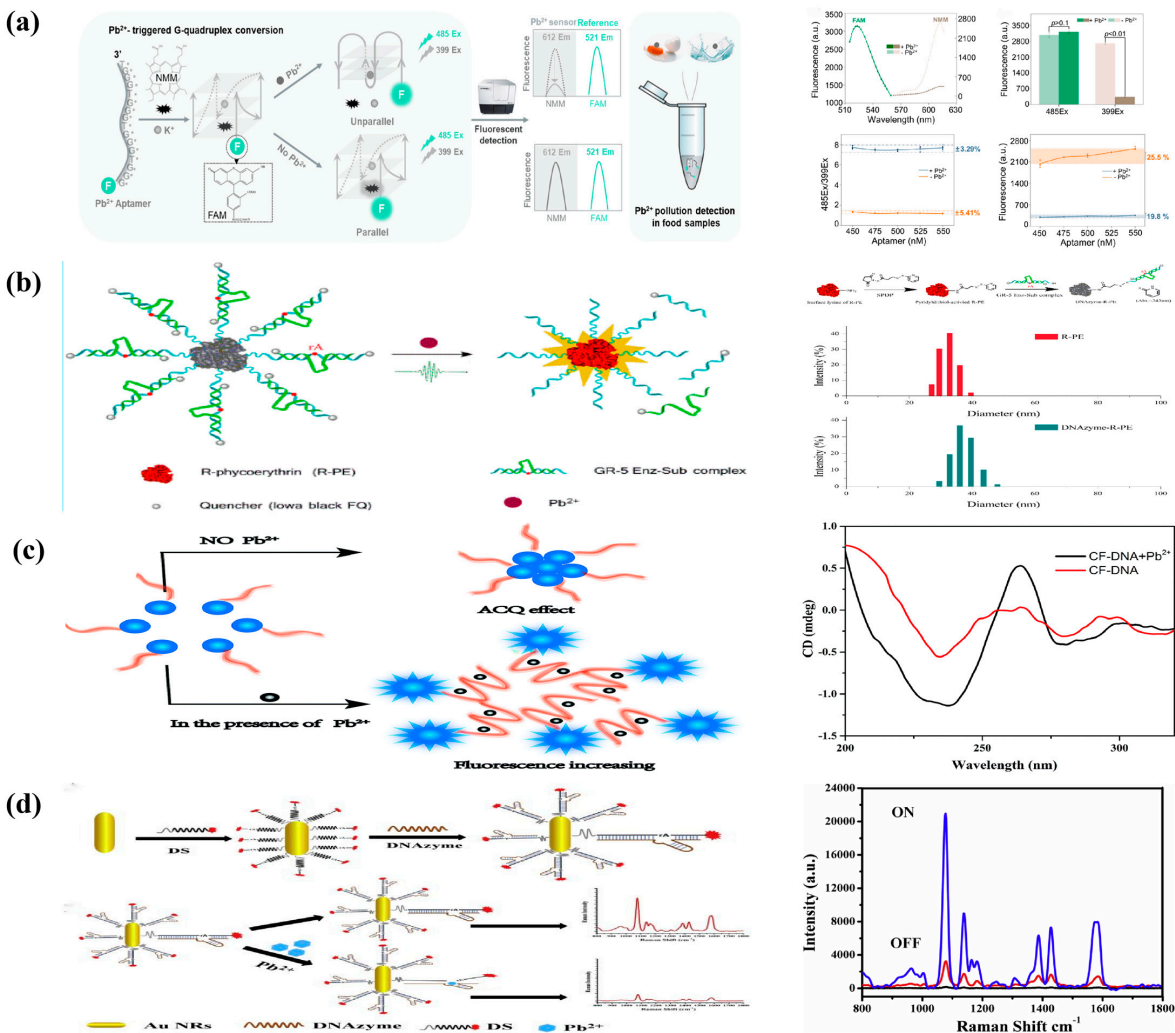
(**a**) Working principle of the aptasensor and its application for Pb^2+^ pollution detection. Fluorescent spectra of the aptasensor. Robustness of the G-quadruplex probes. Corresponding fluorescent intensity [138]. Reprinted with permission [138]. Copyright 2021, Biosensors. (**b**) The sensing strategy of DNAzyme-R-PE for Pb^2+^ detection. The reaction process of the preparation of DNAzyme-R-PE. Dynamic light scattering (DLS) spectra of R-PE and DNAzyme-R-PE [139]. Reprinted with permission [139]. Copyright 2019, Sensors. (**c**) Schematic illustration of analytical method for detecting Pb^2+^ by utilizing the CF-DNA probe. Circular dichroism spectra of CF-DNA (5 mM) in the absence and presence of Pb^2+^ (5 mM) [140]. Reprinted with permission [140]. Copyright 2018, RSC advances. (**d**) Fabrication of the DNAzyme-embedded gold nanorods. The principle of the SERS detection for lead ions via DNAzyme SERS biosensor. SERS platform in the state of ‘on’ (blue curve) and ‘off’ (red curve), and SERS spectra of AuNRs (black curve) [141]. Reprinted with permission [141]. Copyright 2019, Analytica Chimica Acta: X.

Pb^2+^ can selectively react with G-rich single-stranded DNA to induce a conformational shift to a G-quadruplex [132,133] consisting of a planar stack of four guanines supported by Hoogsteen hydrogen bonds. The G-quadruplex is a secondary nucleic acid structure that is not classical [134]. Metal ions can improve the stability of G-quadruplexes, and potassium ions (K^+^) can cause parallel G-quadruplex conformations to develop [135]. Porphyrins, such as NMM, can bind to G-quadruplexes and boost fluorescence upon binding [136]. As a result, NMM can be employed as a luminous indicator of G-quadruplex existence. In addition to Pb^2+^, parallel G-quadruplexes are driven to form non-parallel structures [137]. The cavity within the G-quadruplex shrinks and no longer binds to the NMM, resulting in dramatically diminished NMM fluorescence. As a result, NMM can be used to detect the presence of Pb^2+^. Liu provides a long-lasting and sensitive ratiometric fluorescence technique for detecting Pb^2+^ in food samples (Figure 8a). To detect the presence of Pb^2+^, the G-quadruplex-specific dye NMM was used. Chemically labeled 6-carboxyfluorescein (FAM) was used as a reference to contribute sequentially to the ratiometric Pb^2+^ assay. K^+^ has the property of stabilizing G-quadruplexes. When K^+^ is added, the linear aptamer naturally folds into a parallel structure, permitting the binding and opening of the NMM. The inclusion of Pb^2+^ immediately converts the nucleic acid aptamer into a non-parallel G-quadruplex structure, releasing the NMM dye and drastically reducing its fluorescence. As a result, fluorescence measurements of NMM can be used to monitor the amount of Pb^2+^ [138].

Wu et al. presented a new fluorescent biosensor for Pb^2+^ analysis, DNAzyme-functionalized R-phycoerythrin (DNAzyme-R-PE) (Figure 8b). A deoxyribonuclease substrate complex modified by Iowa-black FQ was immobilized on the surface of SPDP-functionalized R-PE to create the biosensor. The biosensor produced very little fluorescence signal in the absence of Pb^2+^. However, Pb^2+^ recognition can cause substrate cleavage, resulting in R-PE fluorescence recovery. Pb^2+^ was determined using fluorescence change, which has a detection limit of 0.16 nM and a linear range of 0.5–75 nM. R-PE (phycoerythrin) is a stable and inexpensive fluorescent protein obtained from red algae. It emits a strong red-orange fluorescence and has a very high absorption coefficient and quantum yield [139].

Li et al. created a fluorescent sensor for burst-free Pb^2+^ detection by combining a G-rich DNA sequence as a recognition probe with fluorescein (CF) (Figure 8c). The hydrophobic fluorescent units of the probe combine in aqueous solution to create a core with a hydrophilic DNA shell, causing a fluorescence burst due to the aggregation-caused quenching (ACQ) phenomenon. The CF-DNA probe binds to Pb^2+^ and promotes conformational changes to create a quadruple structure in the presence of Pb^2+^. Fluorescence is increased when the CF aggregates are disrupted. Furthermore, fluorescent probes for Pb^2+^ detection have high sensitivity and selectivity, making them suitable for environmental monitoring [140].

Xu et al. created a unique ‘off’ SERS platform by combining AuNRs and deoxyribonuclease-DNA methods for ultra-sensitive and selective Pb^2+^ detection (Figure 8d). As the signal probe, a thio-30-Cy3-labeled probe (SDNA) was employed, and the probe was built on gold nanoparticles via Au-S bonds. Second, the closeness of AuNRs to Cy3-labeled probes resulted in DNA enzymes that produce significant SERS signals when hybridized with AuNRs-modified probes. In the presence of Pb^2+^, the Cy3-labeled probe and AuNR begin to disintegrate through catalytic cleavage of the DNA molecule, resulting in substrate rupture and the formation of a significantly weak SERS signal. The probe in the EDNA-SDNA double-stranded body is hydrolyzed with the help of Pb^2+^, releasing the deoxyribonuclease to allow the other probe to be cleaved. In this instance, the AuNRs separate from the SDNA, considerably lowering the SDNA’s Raman intensity. A very small amount of EDNA can cause the SDNA chain to break on the AuNR surface during the reaction [141].

### 4.4. Cadmium

**Figure 9:**
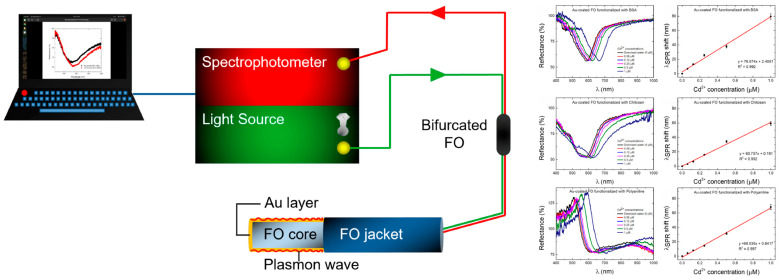
Schematic diagram of the portable FO-SPR sensing system. SPR spectra for varying concentrations from 0 to 1 μM of Cd^2+^ using BSA, chitosan, and PANI/Au-coated FO-SPR sensors. Corresponding calibration curves of the FO-SPR sensors [142]. Reprinted with permission [142]. Copyright 2022, Biosensors.

For the sensitive detection of cadmium ions in water, Olomonea et al. constructed a gold-coated reflecting optical fiber surface plasmon resonance (Au-coated FO-SPR) sensor (Figure 9) and functionalized it with bovine serum albumin (BSA) and polyaniline (PANI) separately. The three sensor functionalization techniques were then assessed and contrasted individually. The BSA-functionalized FO-SPR sensor stands out among these methods for its great sensitivity and low limit of detection (LOD) for the detection of Cd^2+^ at the nanoscale level. Outside of lab facilities, this sensor can be successfully employed for environmental monitoring and quality control of drinking water [142].

### 4.5. Chromium

**Figure 10:**
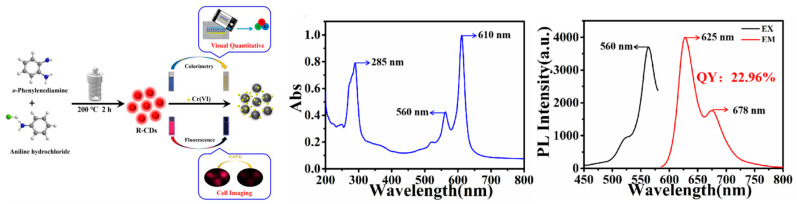
The solid-phase synthesis of R-CDs and its applications in dual-mode Cr(VI) detection and cell imaging. The UV-visible absorption, excitation, and emission spectrum of R-CDs [143]. Reprinted with permission [143]. Copyright 2022, Biosensors.

Red fluorescent carbon dots (R-CDs) offer a new method for the efficient analysis of Cr(VI) in environmental and biological materials due to their superior optical characteristics and biocompatibility. A novel R-CD was created by Hu et al. based on a straightforward green solid-phase production method (Figure 10). Bright-red light with a wavelength of 625 nm is produced by synthetic R-CDs, which also significantly absorbs visible light. The R-CDs in aqueous solution absorption signal major manifestations of Cr(VI) in the absorption and fluorescence signals of R-CDs in aqueous solution are discoloration and fluorescence burst. This is a straightforward procedure for producing R-CDs in solid form from o-phenylenediamine and aniline hydrochloride. Because the process just takes two hours and does not require any solvents, it is in line with green chemistry and further supports the usage of CDs in everyday life. With the addition of Cr(VI), the solution changed from blue to light yellow, and the fluorescence intensity of R-CDs decreased. Several experiments suggest that the unique oxidation properties of Cr(VI) play an important role in the reaction between R-CDs and Cr(VI) [143].

## 5. Conclusions

In general, there are three ways that DNA and metal ions might interact: (i) through heavy-metal-ion-based exchange of hydrogen atoms of Watson–Crick base pairs; (ii) through heavy-metal-ion binding to DNA that is reversible; and (iii) through DNA and HM ions [144]. Sustained cross-linking results in the formation of kinetically inactive compounds. Metal ion-specific DNA is the most thymine (T)-rich sequence, which is highly selective for metal ions and promotes the formation of strong metal-based complexes, specifically T-Hg^2+^-T [145], G-base rich DNA strands, which self-associate into atypical secondary structures called G-quadruplex (G4) [146].

Monitoring and measurement of metals in various settings or in humans has received a lot of interest in recent decades. Because heavy metal accumulation in environmental systems endangers human health, metal levels must be monitored and controlled. To attain this goal, researchers from several academic disciplines collaborated to develop novel tactics. Many different types of metal sensors have been produced, including electrochemical and photochemical sensors. Although metal sensors are based on various technologies, they all have comparable functioning processes. We cover the current state of the art for monitoring heavy-metal ions in this study, with a focus on metal sensors based on biological systems. Although we are unable to discuss all known metal sensors in this review, we hope that it will contribute to a better knowledge of the current state of metal sensors and encourage fresh ideas for developing new ways to generate new metal sensors. We anticipate that metal sensors’ future development will bridge the gap between the laboratory and the ambient domains.

## 6. Current Limitations and Future Prospective

Sensor technology is a very promising detection tool. Some of these sensors are already on the market and others are expected to be in the near future. However, the main bottlenecks currently limiting their practical use are the cumbersome preparation process, poor reproducibility, stability, and sensitivity. On the one hand, the cumbersome synthesis step has been used in many existing studies, and although this method is very sensitive and specific for the detection of heavy-metal ions, it is cumbersome to handle and difficult for meeting the requirements of practical applications. In recent years, advances in nanomaterials processing technology have led to significant improvements in some time-consuming and labor-intensive processes. In addition, some substances are harmful to the environment. When produced and accumulated in large quantities, they can lead to contamination of water resources. Health and environmental issues need to be considered before mass production. Furthermore, characteristics such as simplicity of equipment and ease of use are among the main advantages of biosensor technology currently used in everyday life. As a result, smarter, more convenient, and more portable flexible devices are now taking center stage. This means that future biosensor development will be lighter, easier to manufacture, and smarter.

Many existing sensors suffer from low repeatability, low stability, and low sensitivity. This is a major challenge. Therefore, research into new and efficient methods for detecting heavy metals is urgently needed. Relevant research based on these methods will contribute to human health and safety.

## Data Availability

Not applicable.

## References

[1] March G., Nguyen T.D., Piro B. (2015). Modified electrodes used for electrochemical detection of metal ions in environmental analysis. Biosensors.

[2] Domínguez-Renedo O., Alonso-Lomillo M.A., Arcos-Martínez M.J. (2013). Determination of metals based on electrochemical biosensors. Crit. Rev. Environ. Sci. Technol..

[3] Li M., Gou H., Al-Ogaidi I., Israa, Wu N. (2013). Nanostructured sensors for detection of heavy metals: A review. ACS Sustain. Chem. Eng..

[4] Hussain B., Abbas Y., Ali H., Zafar M., Ali S., Ashraf M.N., Zehra Q., Esinoza S.T.L., Valderrama J.R.D. (2022). Metal and metalloids speciation, fractionation, bioavailability, and transfer toward plants. Metals Metalloids Soil Plant Water Systems.

[5] Bodo M., Balloni S., Lumare E., Bacci M., Calvitti M., Dell’Omo M., Murgia N., Marinucci L. (2010). Effects of sub-toxic cadmium concentrations on bone gene expression program: Results of an in vitro study. Toxicol. In Vitro.

[6] Aragay G., Pons J., Merkoçi A. (2011). Recent trends in macro-, micro-, and nanomaterial-based tools and strategies for heavy-metal detection. Chem. Rev..

[7] Kang H., Lin L., Rong M., Chen X. (2014). A cross-reactive sensor array for the fluorescence qualitative analysis of heavy metal ions. Talanta.

[8] Zaynab M., Al-Yahyai R., Ameen A., Ameen A., Sharif Y., Ali L., Fatima M., Ali Khan K., Li S. (2022). Health and environmental effects of heavy metals. J. King Saud Univ.-Sci..

[9] Zhang W., Zhang P., Liang Y., Chen W., Li L., Wang H., Yu Z., Liu Y., Zhang X. (2022). Rapid electrochemical quantification of trace Hg^2+^ using a hairpin DNA probe and quantum dot modified screen-printed gold electrodes. RSC Adv..

[10] Centers for Disease Control (US) (1991). Preventing Lead Poisoning in Young Children: A Statement.

[11] Garza A., Vega R., Soto E. (2006). Cellular mechanisms of lead neurotoxicity. Med. Sci. Monit..

[12] Turner A. (2019). Cadmium pigments in consumer products and their health risks. Sci. Total Environ..

[13] Arain M.B., Ali I., Yilmaz E., Soylak M. (2018). Nanomaterial’s based chromium speciation in environmental samples: A review. TrAC Trends Anal. Chem..

[14] Jin W., Yan K. (2015). Recent advances in electrochemical detection of toxic Cr (VI). RSC Adv..

[15] Gumpu M.B., Sethuraman S., Krishnan U.M., Rayappan J.B.B. (2015). A review on detection of heavy metal ions in water—An electrochemical approach. Sens. Actuators B Chem..

[16] Aarthy M., Rajesh T., Thirunavoukkarasu M. (2020). Critical review on microbial fuel cells for concomitant reduction of hexavalent chromium and bioelectricity generation. J. Chem. Technol. Biotechnol..

[17] Thevenot D.R., Toth K., Durst R.A., Wilson G.S. (1999). Electrochemical biosensors: Recommended definitions and classification. Pure Appl. Chem..

[18] Mehta J., Bhardwaj S.K., Bhardwaj N., Bhardwaj N., Paul A.K., Kumar P., Kim K.-H., Deep A. (2016). Progress in the biosensing techniques for trace-level heavy metals. Biotechnol. Adv..

[19] Liao J.-M., Yang H., Sun P., Gong J., Wu Q., Wu J. (2022). A review of biosensor development research. China High-Tech..

[20] Youxue W., Meijiao W., Yachen T., Zheng W., Liang G., Cheng L., Shuiqin F., Qing L. (2021). Advances in biosensor research for Salmonella detection. Food Sci..

[21] Ma L.-P., Mao B., Liu B., Li G., Han G., Liu G. (2009). Current status and development trend of biosensor applications. Sens. Microsyst..

[22] Peña-Bahamonde J., Nguyen H.N., Fanourakis S.K., Rodrigues D.F. (2018). Recent advances in graphene-based biosensor technology with applications in life sciences. J. Nanobiotechnol..

[23] Durkalec M., Szkoda J., Kolacz R., Opalinski Z., Zmudzki J. (2015). Bioaccumulation of lead, cadmium and mercury in roe deer and wild boars from areas with different levels of toxic metal pollution. Int. J. Environ. Res..

[24] Evans E.H., Day J.A., Palmer C.D., Price W.J., Smith C.M.M., Tyson J.F. (2005). Atomic spectrometry update. Advances in atomic emission, absorption and fluorescence spectrometry, and related techniques. J. Anal. Atomic Spectrom..

[25] Montes-Bayón M., DeNicola K., Caruso J.A. (2003). Liquid chromatography–inductively coupled plasma mass spectrometry. J. Chromatogr. A.

[26] Zhang Y., Adeloju S.B. (2015). Coupling of non-selective adsorption with selective elution for novel in-line separation and detection of cadmium by vapour generation atomic absorption spectrometry. Talanta.

[27] Pujol L., Evrard D., Groenen-Serrano K., Freyssinier M., Ruffien-Cizsak A. (2014). Electrochemical sensors and devices for heavy metals assay in water: The French groups’ contribution. Front. Chem..

[28] Sadak O. (2023). Chemical sensing of heavy metals in water. Advanced Sensor Technology.

[29] Ullah N., Mansha M., Khan I., Qurashi A. (2018). Nanomaterial-based optical chemical sensors for the detection of heavy metals in water: Recent advances and challenges. TrAC Trends Anal. Chem..

[30] Damborský P., Švitel J., Katrlík J. (2016). Optical biosensors. Essays Biochem..

[31] Gibi C., Liu C.H., Barton S.C., Wu J.J. (2022). Recent Progress in Morphology-Tuned Nanomaterials for the Electrochemical Detection of Heavy Metals. Nanomaterials.

[32] Xie X. (2019). Research on Electrochemical Biosensors Based on Metal Nanomaterials and Biomagnification Technology. Master’s Thesis.

[33] Smart A., Crew A., Pemberton R., Hughes G., Doran O., Hart J.P. (2020). Screen-printed carbon based biosensors and their applications in agri-food safety. TrAC Trends Anal. Chem..

[34] Hughes G., Westmacott K., Honeychurch K.C., Adrian C., Roy P., John H. (2016). Recent advances in the fabrication and application of screen-printed electrochemical (bio) sensors based on carbon materials for biomedical, agri-food and environmental analyses. Biosensors.

[35] Brisset H., Briand J.F., Barry-Martinet R., Duong R., Hy T., Pierre F., Frédéric G., Philippe L., Christine B. (2018). 96X screen-printed gold electrode platform to evaluate electroactive polymers as marine antifouling coatings. Anal. Chem..

[36] Zribi R., Maalej R., Messina E., Gillibert R., Neri G. (2020). Exfoliated 2D-MoS2 nanosheets on carbon and gold screen printed electrodes for enzyme-free electrochemical sensing of tyrosine. Sens. Actuators B Chem..

[37] Motia S., Bouchikhi B., Llobet E., El Bari N. (2020). Synthesis and characterization of a highly sensitive and selective electrochemical sensor based on molecularly imprinted polymer with gold nanoparticles modified screen-printed electrode for glycerol determination in wastewater. Talanta.

[38] Ono A., Togashi H. (2004). Highly selective oligonucleotide-based sensor for mercury (II) in aqueous solutions. Angew. Chem..

[39] Liu T., Chu Z., Jin W. (2019). Electrochemical mercury biosensors based on advanced nanomaterials. J. Mater. Chem. B.

[40] Deshmukh K., Ahamed M.B., Deshmukh R.R., Pasha S., Bhagat P.R., Chidambaram K. (2017). Biopolymer composites with high dielectric performance: Interface engineering. Biopolymer Composites in Electronics.

[41] Frost S.J., Mawad D., Higgins M.J., Ruprai H., Lauto A. (2016). Gecko-inspired chitosan adhesive for tissue repair. NPG Asia Mater..

[42] Do J.S., Lin K.H. (2016). Kinetics of urease inhibition-based amperometric biosensors for mercury and lead ions detection. J. Taiwan Inst. Chem. Eng..

[43] Luo Y.C., Do J.S. (2004). Urea biosensor based on PANi (urease)-Nafion^®^/Au composite electrode. Biosens. Bioelectron..

[44] Saenchoopa A., Klangphukhiew S., Somsub R., Talodthaisong C., Patramanon R., Daduang J., Daduang S., Kulchat S. (2021). A disposable electrochemical biosensor based on screen-printed carbon electrodes modified with silver nanowires/hpmc/chitosan/urease for the detection of mercury (ii) in water. Biosensors.

[45] Cao X., Yue L., Lian F., Wang C., Cheng B., Lv J., Wang Z. (2021). CuO nanoparticles doping recovered the photocatalytic antialgal activity of graphitic carbon nitride. J. Hazard. Mater..

[46] Zhang X., Yang P. (2021). The edge-epitaxial growth of yellow g-C_3_N_4_ on red g-C_3_N_4_ nanosheets with superior photocatalytic activities. Chem. Commun..

[47] Qian X., Meng X., Sun J., Jiang L., Zhu J. (2019). Salt-assisted synthesis of 3D porous g-C_3_N_4_ as a bifunctional photo-and electrocatalyst. ACS Appl. Mater. Interfaces.

[48] Wu J., Tian L., Duan H., Cheng Y., Shi L. (2021). Unveiling the working mechanism of g-C_3_N_4_ as a protection layer for lithium-and sodium-metal anode. ACS Appl. Mater. Interfaces.

[49] Jin Y., Kang Q., Guo X., Zhang B., Shen D., Zou G. (2018). Electrochemical-signal-amplification strategy for an electrochemiluminescence immunoassay with g-C_3_N_4_ as tags. Anal. Chem..

[50] Huang D., Li Z., Zeng G., Zou C., Xue W., Gong X., Yan X., Chen S., Wang W., Cheng M. (2019). Megamerger in photocatalytic field: 2D g-C_3_N_4_ nanosheets serve as support of 0D nanomaterials for improving photocatalytic performance. Appl. Catal. B Environ..

[51] Xu Y., Wen Z., Wang T., Zhang M., Ding C., Guo Y., Jiang D., Wang K. (2020). Ternary Z-scheme heterojunction of Bi SPR-promoted BiVO_4_/g-C_3_N_4_ with effectively boosted photoelectrochemical activity for constructing oxytetracycline aptasensor. Biosens. Bioelectron..

[52] Zhang L., Feng L., Li P., Chen X., Wang H. (2021). Near-infrared light-driven photoelectrochemical sensor for mercury (II) detection using bead-chain-like Ag@Ag_2_S nanocomposites. Chem. Eng. J..

[53] Xiao F.X., Liu B. (2018). Plasmon-Dictated Photo-Electrochemical Water Splitting for Solar-to-Chemical Energy Conversion: Current Status and Future Perspectives. Adv. Mater. Interfaces.

[54] Wang D., Ding Z., Zhou H., Chen L., Feng X. (2021). Au Nanoparticle-Decorated TiO_2_ Nanowires for Surface Plasmon Resonance-Based Photoelectrochemical Bioassays with a Solid–Liquid–Air Triphase Interface. ACS Appl. Nano Mater..

[55] Xu Y., Jiang D., Zhang M., Zhang Z., Wang K. (2020). High-performance photoelectrochemical aptasensor for enrofloxacin based on Bi-doped ultrathin polymeric carbon nitride nanocomposites with SPR effect and carbon vacancies. Sens. Actuators B Chem..

[56] Li M., Wu Y., An S., Yan Z. (2022). Au NP-Decorated g-C_3_N_4_-Based Photoelectochemical Biosensor for Sensitive Mercury Ions Analysis. ACS Omega.

[57] Hasanjani H.R.A., Zarei K. (2019). An electrochemical sensor for attomolar determination of mercury (II) using DNA/poly-L-methionine-gold nanoparticles/pencil graphite electrode. Biosens. Bioelectron..

[58] Ma J. (2021). Preparation of Gold Nanobiosensor and Its Application in Heavy Metal Detection. Master’s Thesis.

[59] Narouei F.H., Livernois L., Andreescu D., Andreescu S. (2021). Highly sensitive mercury detection using electroactive gold-decorated polymer nanofibers. Sens. Actuators B Chem..

[60] Atapour M., Amoabediny G., Ahmadzadeh-Raji M. (2019). Integrated optical and electrochemical detection of Cu^2+^ ions in water using a sandwich amino acid–gold nanoparticle-based nano-biosensor consisting of a transparent-conductive platform. RSC Adv..

[61] Qian Z.S., Shan X.Y., Chai L.J., Chen J.R., Hui F. (2015). A fluorescent nanosensor based on graphene quantum dots–aptamer probe and graphene oxide platform for detection of lead (II) ion. Biosens. Bioelectron..

[62] Tang Y., Hu H., Zhang M.G., Song J., Nie L., Wang S., Niu G., Huang P., Lu G., Chen X. (2015). An aptamer-targeting photoresponsive drug delivery system using “off–on” graphene oxide wrapped mesoporous silica nanoparticles. Nanoscale.

[63] Yang D., Liu X., Zhou Y., Lin L., Lin T. (2017). Aptamer-based biosensors for detection of lead (ii) ion: A review. Anal. Methods.

[64] Zhu N., Liu X., Peng K., Cao H., Yuan M., Ye T., Wu X., Yin F., Yu J., Hao L. (2022). A novel aptamer-imprinted polymer-based electrochemical biosensor for the detection of lead in aquatic products. Molecules.

[65] Ding J., Zhang D., Liu Y., Zhan X., Lu Y., Zhou P., Zhang D. (2020). An electrochemical aptasensor for Pb^2+^ detection based on metal–organic-framework-derived hybrid carbon. Biosensors.

[66] Yang S., Liu P., Wang Y., Guo Z., Qu L. (2020). Electrochemical sensor using poly-(l-cysteine) functionalized CuO nanoneedles/N-doped reduced graphene oxide for detection of lead ions. RSC Adv..

[67] Gao L., Deng Y., Liu H., Solomon K., Zhang B., Cai H. (2022). Detection of Pb^2+^ in tea using aptamer labeled with AIEgen nanospheres based on MOFs sensors. Biosensors.

[68] Tsai Y.C., Chen S.Y., Liaw H.W. (2007). Immobilization of lactate dehydrogenase within multiwalled carbon nanotube-chitosan nanocomposite for application to lactate biosensors. Sens. Actuators B Chem..

[69] Hamdy M.E., Del Carlo M., Hussein H.A., Salah T.A., El-Dee A.H., Emara M.M., Pezzoni G., Compagnone D. (2018). Development of gold nanoparticles biosensor for ultrasensitive diagnosis of foot and mouth disease virus. J. Nanobiotechnol..

[70] Jiang P., Wang Y., Zhao L., Ji C., Chen D., Nie L. (2018). Applications of gold nanoparticles in non-optical biosensors. Nanomaterials.

[71] Kang X., Mai Z., Zou X., Cai P., Mo J. (2007). A novel glucose biosensor based on immobilization of glucose oxidase in chitosan on a glassy carbon electrode modified with gold–platinum alloy nanoparticles/multiwall carbon nanotubes. Anal. Biochem..

[72] Rabai S., Teniou A., Catanante G., Benounis M., Marty J.-L., Rhouati A. (2021). Fabrication of AuNPs/MWCNTS/chitosan nanocomposite for the electrochemical aptasensing of cadmium in water. Sensors.

[73] Attaallah R., Amine A. (2022). An ultrasensitive and selective determination of cadmium ions at ppt level using an enzymic membrane with colorimetric and electrochemical detection. Biosensors.

[74] Liu Y., Zhang D., Ding J., Hayat K., Yang X., Zhan X., Zhang D., Lu Y., Zhou P. (2020). Label-free and sensitive determination of cadmium ions using a Ti-modified Co_3_O_4_-based electrochemical aptasensor. Biosensors.

[75] Meng Q., Zhang B., Fan L., Liu H., Sun L. (2019). Efficient BiVO_4_ photoanodes by postsynthetic treatment: Remarkable improvements in photoelectrochemical performance from facile borate modification. Angew. Chem..

[76] Lu H., Andrei V., Jenkinson K.J., Regoutz A., Li N., Creissen C.E., Wheatley A.E.H., Hao H., Reisner E., Wright D.S. (2018). Single-Source Bismuth (Transition Metal) Polyoxovanadate Precursors for the Scalable Synthesis of Doped BiVO_4_ Photoanodes. Adv. Mater..

[77] Phanichphant S., Nakaruk A., Chansaenpak K., Channei D. (2019). Evaluating the photocatalytic efficiency of the BiVO_4_/rGO photocatalyst. Sci. Rep..

[78] Wu W., Tan Z., Chen X., Chen X., Cheng L., Wu H., Li P., Zhang Z. (2022). Carnation-like morphology of BiVO_4_-7 enables sensitive photoelectrochemical determination of Cr(VI) in the food and environment. Biosensors.

[79] Juang Y.J., Li W.S., Chen P.S. (2017). Fabrication of microfluidic paper-based analytical devices by filtration-assisted screen printing. J. Taiwan Inst. Chem. Eng..

[80] Amine A., Arduini F., Moscone D., Palleschi G. (2016). Recent advances in biosensors based on enzyme inhibition. Biosens. Bioelectron..

[81] Dabhade A., Jayaraman S., Paramasivan B. (2021). Development of glucose oxidase-chitosan immobilized paper biosensor using screen-printed electrode for amperometric detection of Cr (VI) in water. 3 Biotech.

[82] Bowers M.T., Liu D., Wyttenbach T. (2008). Interaction of divalent metal ions with the hormone oxytocin: Hormone receptor binding. J. Am. Chem. Soc..

[83] Liu D., Seuthe A.B., Ehrler O.T., Zhang X., Wyttenbach T., Hsu J.F., Bowers M.T. (2005). Oxytocin-receptor binding: Why divalent metals are essential. J. Am. Chem. Soc..

[84] Attia J., Nir S., Mervinetsky E., Balogh D., Gitlin-Domagalska A., Alshanski I., Reches M., Hurevich M., Yitzchaik S. (2021). Non-covalently embedded oxytocin in alkanethiol monolayer as Zn^2+^ selective biosensor. Sci. Rep..

[85] Khan A.U., Zhao S., Liu G. (2016). Key parameter controlling the sensitivity of plasmonic metal nanoparticles: Aspect ratio. J. Phys. Chem. C.

[86] Hou J., Li M., Song Y. (2016). Recent advances in photonic crystal sensors. Sci. Sin. Chim..

[87] Lee B. (2003). Review of the present status of optical fiber sensors. Opt. Fiber Technol..

[88] Shang L., Dong S., Nienhaus G.U. (2011). Ultra-small fluorescent metal nanoclusters: Synthesis and biological applications. Nano Today.

[89] Saleh S.M., Ali R., Hirsch T., Wolfbeis O.S. (2011). Detection of biotin–avidin affinity binding by exploiting a self-referenced system composed of upconverting luminescent nanoparticles and gold nanoparticles. J. Nanopart. Res..

[90] Srivastava P., Razi S.S., Ali R., Gupta R.C., Yadav S.S., Narayan G., Misra A. (2014). Selective naked-eye detection of Hg^2+^ through an efficient turn-on photoinduced electron transfer fluorescent probe and its real applications. Anal. Chem..

[91] Razi S.S., Ali R., Gupta R.C., Dwivedi S.K., Sharma G., Koch B., Misra A. (2016). Phenyl-end-capped-thiophene (PT type) based ICT fluorescent probe (D–π–A) for detection of Hg^2+^ and Cu^2+^ ions: Live cell imaging and logic operation at molecular level. J. Photochem. Photobiol. A Chem..

[92] Srivastava P., Shahid M., Misra A. (2011). Protein assisted fluorescence enhancement of a dansyl containing fluorescent reagent: Detection of Hg^+^ ion in aqueous medium. Org. Biomol. Chem..

[93] Kim I., Lee N.E., Jeong Y.J., Chung Y.H., Cho B.K., Lee E. (2014). Micellar and vesicular nanoassemblies of triazole-based amphiphilic probes triggered by mercury (II) ions in a 100% aqueous medium. Chem. Commun..

[94] Zong L., Wang C., Song Y., Xie Y., Zhang P., Peng Q., Li Q., Li Z. (2017). A dual-function probe based on naphthalene diimide for fluorescent recognition of Hg^2+^ and colorimetric detection of Cu^2+^. Sens. Actuators B Chem..

[95] Fang W., Zhang G., Chen J., Kong L., Yang L., Bi H., Yang J. (2016). An AIE active probe for specific sensing of Hg^2+^ based on linear conjugated bis-Schiff base. Sens. Actuators B Chem..

[96] Aliberti A., Vaiano P., Caporale A., Consales M., Ruvo M., Cusano A. (2017). Fluorescent chemosensors for Hg^2+^ detection in aqueous environment. Sens. Actuators B Chem..

[97] Zhong K., Zhou X., Hou R., Zhou P., Hou S., Bian Y., Zhang G., Tang L., Shang X. (2014). A water-soluble highly sensitive and selective fluorescent sensor for Hg^2+^ based on 2-(2-(8-hydroxyquinolin)-yl) benzimidazole via ligand-to-metal charge transfer (LMCT). RSC Adv..

[98] Wu Z., Zhang Y., Ma J.S., Yang G. (2006). Ratiometric Zn^2+^ sensor and strategy for Hg^2+^ selective recognition by central metal ion replacement. Inorg. Chem..

[99] Rani B.K., John S.A. (2018). Fluorogenic mercury ion sensor based on pyrene-amino mercapto thiadiazole unit. J. Hazard. Mater..

[100] Moon S.Y., Youn N.J., Park S.M., Chang S.K. (2005). Diametrically disubstituted cyclam derivative having Hg^2+^-selective fluoroionophoric behaviors. J. Org. Chem..

[101] Lee M.H., Lee S.W., Kim S.H., Kang C., Kim J.S. (2009). Nanomolar Hg (II) detection using Nile blue chemodosimeter in biological media. Org. Lett..

[102] Ding L., Wu M., Li Y., Chen Y., Su J. (2014). New fluoro-and chromogenic chemosensors for the dual-channel detection of Hg^2+^ and F^−^. Tetrahedron Lett..

[103] Song K.C., Kim J.S., Park S.M., Chung K.C., Ahn S., Chang S.K. (2006). Fluorogenic Hg^2+^-selective chemodosimeter derived from 8-hydroxyquinoline. Org. Lett..

[104] Angupillai S., Hwang J.Y., Lee J.Y., Rao B.A., Son Y.A. (2015). Efficient rhodamine-thiosemicarbazide-based colorimetric/fluorescent ‘turn-on’ chemodosimeters for the detection of Hg^2+^ in aqueous samples. Sens. Actuators B Chem..

[105] Madhu S., Kumar S., Chatterjee T., Ravikanth M. (2014). Synthesis, X-ray structure, spectral and electrochemical properties of a β-meso covalently linked BODIPY–Ru (II) dipyrrin complex. New J. Chem..

[106] Gao Y., Ma T., Ou Z., Cai W., Yang G., Li Y., Xu M., Li Q. (2018). Highly sensitive and selective turn-on fluorescent chemosensors for Hg^2+^ based on thioacetal modified pyrene. Talanta.

[107] Yang Y., Shen R., Wang Y.Z., Qiu F.Z., Feng Y., Tang X.L., Bai D., Zhang G.L., Liu W.S. (2018). A selective turn-on fluorescent sensor for Hg (II) in living cells and tissues. Sens. Actuators B Chem..

[108] Luo J., Xie Z., Lam J.W., Cheng L., Chen H., Qiu C., Kwok H.S., Zhan X., Liu Y., Zhu D. (2001). Aggregation-induced emission of 1-methyl-1, 2, 3, 4, 5-pentaphenylsilole. Chem. Commun..

[109] Tang L., Yu H., Zhong K., Gao X., Li J. (2019). An aggregation-induced emission-based fluorescence turn-on probe for Hg^2+^ and its application to detect Hg^2+^ in food samples. RSC Adv..

[110] Fan M., Pan Z., Wang C., Guo Y., Sun J., Liu M., Peng B., Wu J., Fang Y. (2022). Quantitative visual detection of mercury ions with ratiometric fluorescent test paper sensor. Front. Chem..

[111] Ali R., Ghannay S., Messaoudi S., Alminderej F.M., Aouadi K., Saleh S.M. (2022). A Reversible Optical Sensor Film for Mercury Ions Discrimination Based on Isoxazolidine Derivative and Exhibiting pH Sensing. Biosensors.

[112] Gupta S., Sarkar S., Katranidis A., Bhattacharya J. (2019). Development of a cell-free optical biosensor for detection of a broad range of mercury contaminants in water: A plasmid DNA-based approach. ACS Omega.

[113] Kim Y., Choi H., Shin W.H., Oh J.M., Koo S.M., Kim Y., Lee T., Yu B.J., Park C. (2021). Development of colorimetric whole-cell biosensor for detection of heavy metals in environment for public health. Int. J. Environ. Res. Public Health.

[114] De Acha N., Elosúa C., Arregui F.J. (2020). Development of an aptamer based luminescent optical fiber sensor for the continuous monitoring of Hg^2+^ in aqueous media. Sensors.

[115] Huang D., Liu X., Lai C., Qin L., Zhang C., Yi H., Zeng G., Li B., Deng R., Liu S. (2019). Colorimetric determination of mercury (II) using gold nanoparticles and double ligand exchange. Microchim. Acta.

[116] Tsogas G.Z., Kappi F.A., Vlessidis A.G., Giokas D.L. (2018). Recent advances in nanomaterial probes for optical biothiol sensing: A review. Anal. Lett..

[117] Yoon S.J., Nam Y.S., Lee Y., Oh I.H., Lee K. (2021). A dual colorimetric probe for rapid environmental monitoring of Hg^2+^ and As^3+^ using gold nanoparticles functionalized with d-penicillamine. RSC Adv..

[118] Duan J., Yin H., Wei R., Wang W. (2014). Facile colorimetric detection of Hg^2+^ based on anti-aggregation of silver nanoparticles. Biosens. Bioelectron..

[119] Tolessa T., Tan Z.Q., Yin Y.G., Liu J.F. (2018). Single-drop gold nanoparticles for headspace microextraction and colorimetric assay of mercury (II) in environmental waters. Talanta.

[120] Gu Y., Jiao L., Cao F., Liu X., Zhou Y., Yang C., Gao Z., Zhang M., Lin P., Han Y. (2022). A Real-Time Detection Method of Hg^2+^ in Drinking Water via Portable Biosensor: Using a Smartphone as a Low-Cost Micro-Spectrometer to Read the Colorimetric Signals. Biosensors.

[121] Mao M.X., Zheng R., Peng C.F., Wei X.L. (2020). DNA–Gold Nanozyme-modified paper device for enhanced colorimetric detection of mercury ions. Biosensors.

[122] Long F., Zhu A., Shi H. (2013). Recent advances in optical biosensors for environmental monitoring and early warning. Sensors.

[123] Tan L., Zhang Y., Qiang H., Li Y., Sun J., Hu L., Chen Z. (2016). A sensitive Hg (II) colorimetric sensor based on synergistic catalytic effect of gold nanoparticles and Hg. Sens. Actuators B Chem..

[124] Peng C.F., Pan N., Xie Z.J., Wu L.L. (2016). Highly sensitive and selective colorimetric detection of Hg^2+^ based on the separation of Hg^2+^ and formation of catalytic DNA–gold nanoparticles. Anal. Methods.

[125] Chen X., Sun Y., Mo X., Gao Q., Deng Y., Hu M., Zou J., Nie J., Zhang Y. (2021). On-site, rapid and visual method for nanomolar Hg^2+^ detection based on the thymine–Hg–thymine triggered “double” aggregation of Au nanoparticles enhancing the Tyndall effect. RSC Adv..

[126] Gao L., Lv Q., Xia N., Lin Y., Lin F., Han B. (2021). Detection of mercury ion with high sensitivity and selectivity using a DNA/graphene oxide hybrid immobilized on glass slides. Biosensors.

[127] Sun T., Li X., Jin X., Wu Z., Chen X., Qiu J. (2022). Function of Graphene Oxide as the “Nanoquencher” for Hg^2+^ Detection Using an Exonuclease I-Assisted Biosensor. Int. J. Mol. Sci..

[128] Song X. (2019). Research on Biosensor Based on DNA Isothermal Signal Amplification for Detection of Heavy Metal Ions in Water Environment. Master’s Thesis.

[129] Zhi L., Zhang S., Li M., Tu J., Lu X. (2022). Achieving ultrasensitive point-of-care assay for mercury ions with a triple-mode strategy based on the mercury-triggered dual-enzyme mimetic activities of Au/WO_3_ hierarchical hollow nanoflowers. ACS Appl. Mater. Interfaces.

[130] Ding L., Xu B., Li T., Huang J., Bai W. (2018). A “turn-on” fluorescence copper biosensor based on DNA cleavage-dependent graphene oxide-dsDNA-Cdte quantum dots complex. Sensors.

[131] Chen Y., Pan H., Wang F., Zhao Y., Yin H., Chen Y., Zhang J., Jiang J. (2019). An ultrafast BODIPY single molecular sensor for multi-analytes (acid/base/Cu^2+^/Bi^3+^) with different sensing mechanism. Dyes Pigment..

[132] Wang C., Cheng H., Sun Y., Xu Z., Lin H., Lin Q., Zhang C. (2015). Nanoclusters prepared from a silver/gold alloy as a fluorescent probe for selective and sensitive determination of lead (II). Microchim. Acta.

[133] Xu H., Xu P., Gao S., Zhang S., Zhao X., Fan C., Zuo X. (2013). Highly sensitive recognition of Pb^2+^ using Pb^2+^ triggered exonuclease aided DNA recycling. Biosens. Bioelectron..

[134] Li W., Hou X.M., Wang P.Y., Xi X.G., Li M. (2013). Direct measurement of sequential folding pathway and energy landscape of human telomeric G-quadruplex structures. J. Am. Chem. Soc..

[135] Wu Y., Shi Y., Deng S., Wu C., Deng R., He G., Zhou M., Zhong K., Gao H. (2021). Metal-induced G-quadruplex polymorphism for ratiometric and label-free detection of lead pollution in tea. Food Chem..

[136] Nicoludis J.M., Barrett S.P., Mergny J.L., Yatsunyk L.A. (2012). Interaction of human telomeric DNA with N-methyl mesoporphyrin IX. Nucleic Acids Res..

[137] Li T., Wang E., Dong S. (2009). Potassium—Lead-switched G-quadruplexes: A new class of DNA logic gates. J. Am. Chem. Soc..

[138] Liu Y., Yang H., Wan R., Khan M.R., Wang N., Busquets R., Deng R., He Q., Zhao Z. (2021). Ratiometric G-quadruplex assay for robust lead detection in food samples. Biosensors.

[139] Wu J., Lu Y., Ren N., Jia M., Wang R., Zhang J. (2019). DNAzyme-functionalized R-phycoerythrin as a cost-effective and environment-friendly fluorescent biosensor for aqueous Pb^2+^ detection. Sensors.

[140] Li Q., Jia Y., Feng Z., Liu F. (2018). A highly sensitive and selective fluorescent probe without quencher for detection of Pb^2+^ ions based on aggregation-caused quenching phenomenon. RSC Adv..

[141] Xu W., Zhao A., Zuo F., Hussain H.M.J., Khan R. (2019). A “turn-off” SERS aptasensor based DNAzyme-gold nanorod for ultrasensitive lead ion detection. Anal. Chim. Acta X.

[142] Şolomonea B.G., Jinga L.I., Antohe V.A., Socol G., Antohe I. (2022). Cadmium ions’ trace-level detection using a portable fiber optic—Surface plasmon resonance sensor. Biosensors.

[143] Hu J., Wang X., Wei H., Zhao L., Yao B., Zhang C., Zhou J., Liu J., Yang S. (2022). Solid-Phase Synthesis of Red Fluorescent Carbon Dots for the Dual-Mode Detection of Hexavalent Chromium and Cell Imaging. Biosensors.

[144] Sun C., Ou X., Cheng Y., Zhai T., Liu B., Lou X., Xia F. (2019). Coordination-induced structural changes of DNA-based optical and electrochemical sensors for metal ions detection. Dalton Trans..

[145] Miyake Y., Togashi H., Tashiro M., Yamaguchi H., Oda S., Kudo M., Tanaka Y., Kondo Y., Sawa R., Fujimoto T. (2006). MercuryII-mediated formation of thymine− HgII− thymine base pairs in DNA duplexes. J. Am. Chem. Soc..

[146] Gellert M., Lipsett M.N., Davies D.R. (1962). Helix formation by guanylic acid. Proc. Natl. Acad. Sci. USA.

